# Brain‐Wide Neuroregenerative Gene Therapy Improves Cognition in a Mouse Model of Alzheimer's Disease

**DOI:** 10.1002/advs.202410080

**Published:** 2025-02-14

**Authors:** Zheng Wu, Liang Xu, Yu Xie, Abhijeet Sambangi, Shreya Swaminathan, Zifei Pei, Wenyu Ji, Zeru Li, Yaowei Guo, Zhifei Li, Gong Chen

**Affiliations:** ^1^ State Key Laboratory of Bioactive Molecules and Druggability Assessment Guangdong Basic Research Center of Excellence for Natural Bioactive Molecules and Discovery of Innovative Drugs Key Laboratory of CNS Regeneration (Ministry of Education) Guangdong Key Laboratory of Non‐Human Primate Research GHM Institute of CNS Regeneration Jinan University Guangzhou 510632 China; ^2^ Department of Biology Huck Institutes of Life Sciences Pennsylvania State University University Park PA 16802 USA

**Keywords:** Alzheimer's disease, gene therapy, neurodegeneration, NeuroD1

## Abstract

Alzheimer's disease (AD) is a progressive and irreversible brain disorder with extensive neuronal loss in the neocortex and hippocampus. Current therapeutic interventions focus on the early stage of AD but lack effective treatment for the late stage of AD, largely due to the inability to replenish the lost neurons and repair the broken neural circuits. In this study, by using engineered adeno‐associated virus vectors that efficiently cross the blood–brain‐barrier in the mouse brain, a brain‐wide neuroregenerative gene therapy is developed to directly convert endogenous astrocytes into functional neurons in a mouse model of AD. It is found that ≈500 000 new neurons are regenerated and widely distributed in the cerebral cortex and hippocampus. Importantly, it is demonstrated that the converted neurons can integrate into pre‐existing neural networks and improve various cognitive performances in AD mice. Chemogenetic inhibition of the converted neurons abolishes memory enhancement in AD mice, suggesting a pivotal role for the newly converted neurons in cognitive restoration. Together, brain‐wide neuroregenerative gene therapy may provide a viable strategy for the treatment of AD and other brain disorders associated with massive neuronal loss.

## Graphical abstract

NeuroD1 mediates brain‐wide astrocyte‐to‐neuron gene therapy conversion in 5xFAD mouse brain. Regenerated neurons can fire action potentials and integrate into pre‐existing neural networks, ultimately improving cognition in AD mice.

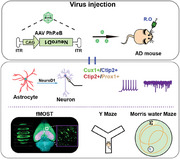

## 1 Introduction

Alzheimer's disease is a devastating neurodegenerative disorder that affects millions of people globally. The pathological hallmarks of AD include the deposition of Aβ plaques and tau‐rich neurofibrillary tangles, which lead to neuroinflammation, oxidative stress, and ultimately, the loss of neurons and synaptic connectivity.^[^ ^1^, ^2^ ^]^ While the exact mechanisms underlying AD progression are still unclear, it is widely accepted that the failure to clear toxic Aβ and tau protein from the brain contributes significantly to disease onset and progression.^[^ ^3^ ^]^ Recent clinical trials aimed at reducing Aβ achieved some success in slowing the progression of AD in early‐stage patients but had little effect on cognitive functions in patients with moderate to severe AD symptoms.^[^ ^4^, ^5^ ^]^ This correlation may be attributed to the substantial neuronal loss observed in the brains of patients with mid‐to‐late AD. Obviously, therapeutic strategies based on the removal of harmful proteins lack the capacity to regenerate neurons. Thus, it is pivotal to search for neuron replacement therapy in order to reverse memory deficits.

Regenerating neurons and repairing degenerated neural networks in AD animals, through either boosting endogenous neurogenesis in the hippocampus^[^ ^6^ ^]^ or transplanting external stem cells,^[^ ^7^ ^]^ bring new possibilities for the treatment of AD. However, exogenous cell transplantation may induce immune rejection and tumorigenesis, leading to potential medical risks. Meanwhile, adult neurogenesis occurs only in specific regions of the mammalian brain, and this ability declines sharply with age increase.^[^ ^8^ ^]^ Furthermore, whether adult neurogenesis exists in the human brain is still controversial.^[^ ^9^, ^10^ ^]^ Alternatively, recently developed astrocyte‐to‐neuron (AtN) conversion has shown great potential for regenerating new neurons in AD animal brains, but whether it can produce any cognitive benefits is still unknown.^[^ ^11^ ^]^ Unlike other neurodegenerative diseases, AD patients' brains show extensive neuronal loss across different brain areas, especially in brain regions highly relevant to learning and memory functions, such as the cortex and hippocampus.^[^ ^12^ ^]^ Therefore, how to achieve brain‐wide neuron regeneration in AD brains represents a significant challenge in the regenerative medicine field.^[^ ^13^ ^]^

In this study, using engineered adeno‐associated virus (AAV) vectors that efficiently cross the blood–brain‐barrier (BBB) in the mouse brain, we developed a brain‐wide neuroregenerative gene therapy through the conversion of endogenous astrocytes into functional neurons in a mouse model of AD. We show that about half a million of new neurons are regenerated and widely distributed in the cerebral cortex and hippocampus. Importantly, the converted neurons can integrate into pre‐existing neural networks and improve various cognitive performances in AD mice. Meanwhile, chemogenetic inhibition of the converted neurons can eliminate memory improvement, suggesting that converted new neurons contribute significantly to cognitive improvement in AD mice. To investigate the mechanism of AtN conversion, we performed single‐cell RNA sequencing (scRNA‐Seq) studies following NeuroD1 initiation of AtN conversion in the hippocampus and found that astrocytic transcriptome was changed toward neuronal transcriptome during the AtN conversion process. Taking advantage of the abundance and wide distribution of resident astrocytes in the brain, our brain‐wide AtN conversion gene therapy sheds new light on the restoration of cognitive functions after AD and may be also applicable to the treatment of other neurological disorders previously considered untreatable.

## 2 Results

### 2.1 Local Neuroregeneration via NeuroD1‐Mediated AtN Conversion in the Hippocampus of 5xFAD Mice

**Figure 1:**
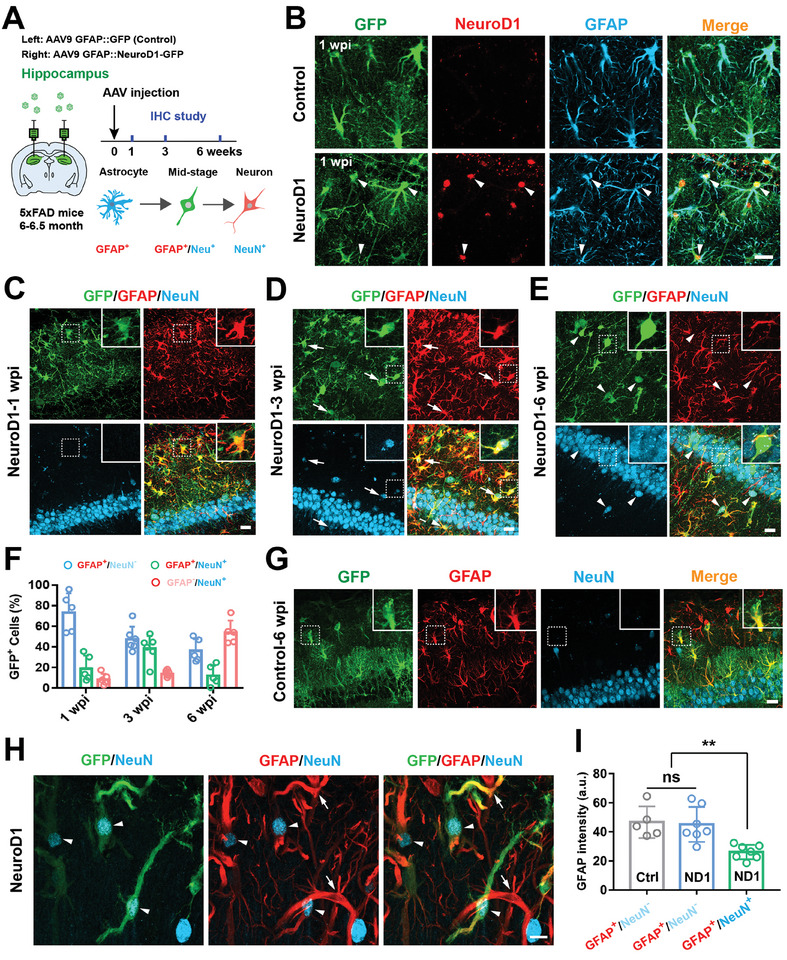
NeuroD1‐mediated local AtN conversion in the hippocampus of 5xFAD mice. A) Schematic diagram showing the experimental design to investigate changes in cell morphology and markers at different time points. The area in green indicates the hippocampus. B) Representative images showing that the control virus transducing GFP^+^ cells (green) co‐localized with the astrocytic markers GFAP (cyan) but not NeuroD1(red) at 1 week after AAV9 injection (top, *n* = 3 mice), and the GFP^+^ cells co‐localized with GFAP and specifically expressing NeuroD1 at 1 wpi of AAV9‐GFAP::NeuroD1‐GFP injection (bottom, *n* = 3 mice). Scale bar, 20 µm. C–E) Representative images illustrating the gradual change from astrocytes to neurons over a 6‐week time window of NeuroD1‐overexpression in hippocampal astrocytes. Note that most GFP^+^ cells co‐labeled with GFAP at early time points after AAV‐NeuroD1 injection (C); 3 weeks after AAV injection, a neuronal marker (NeuN) was obtained while retaining an astrocyte marker (GFAP), these cells were defined as transitional cells (D); but then lost GFAP signal and continued to express NeuN (cyan) signal (E). Dashed boxes represent the enlarged images. Scale bar, 20 µm. F) Quantified data showing the type of the AAV9‐NueroD1 infected cells at 1 week (*n* = 5 mice), 3 weeks (*n* = 6 mice), and 6 weeks (*n* = 5 mice) post injection. Note that the proportion of NeuroD1 overexpressed astrcoytes (GFAP^+^/NeuN^−^) were gradually decreased (blue bar), converted neurons (GFAP^−^/NeuN^+^) were gradually increased (red bar), while the proportion of transitional stage cells (GFAP^+^/NeuN^+^) were first increased and then decreased (green bar). G) All of the GFP^+^ cells were co‐labeled with astrocytic marker GFAP at 6 wpi, no transitional stage of cells and converted neurons were detected in the control group (*n* = 3 mice). Scale bar, 20 µm. H) High‐resolution confocal images showing the transitional stage cells that were observed in the hippocampus during NeuroD1‐mediated AtN conversion. Compared with uninfected astrocytes (arrows, GFP^−^), AAV‐infected cells (GFP^+^) contained clear NeuN signals in the cell body and weaker GFAP (arrowheads). Scale bar, 10 µm. I) Quantified data showing that the GFAP fluorescence intensity in transitional stage cells (GFAP^+^/NeuN^+^, green bar) was significantly decreased compared with their neighboring AAV‐uninfected astrocytes (GFAP^+^/NeuN^−^, blue bar, *n* = 7 mice) or the hippocampal astrocytes in control virus‐treated group (GFAP^+^/NeuN^−^, gray bar, *n* = 5 mice). Data are shown as mean ± SD. **p < 0.01, One‐way ANOVA with Tukey's multiple comparisons test.

The hippocampus is closely related to learning and memory which undergoes significant neuronal loss in AD brains.^[^ ^12^, ^14^ ^]^ We previously reported that NeuroD1 can reprogram cortical reactive astrocytes into functional neurons in mouse brains of 5xFAD and stroke.^[^ ^11^, ^15^ ^]^ Since 5xFAD mice, at 6‐6.5 months of age, show significant pathological phenotypes and cognitive impairment in our previous studies,^[^ ^16^, ^17^ ^]^ we used mice at this age to explore the potential of NeuroD1 mediated AtN conversion in the hippocampus in vivo (**Figure** **1**A). To express NeuroD1 specifically in astrocytes, we employed astrocyte‐specific promoter *Gfap* to directly drive the expression of NeuroD1. AAV9‐GFAP::NeuroD1‐P2A‐GFP were directly injected into 5xFAD mouse hippocampus (6–6.5 months of age), and AAV9‐GFAP::GFP was selected as a control. Then mice were sacrificed at 1 week post injection (wpi), 3 and 6 wpi for immunohistological study (Figure 1A). In the control group, almost all of the GFP^+^ cells were co‐stained by GFAP and lacked of NeuroD1 expression (Figure 1B top). Meanwhile, we observed a strong NeuroD1 immunostaining signal in GFAP^+^ astrocytes at 1 week after AAV9‐GFAP::NeuroD1‐P2A‐GFP injection (Figure 1B bottom). Then we tracked changes in the molecular identity of NeuroD1‐expressing astrocytes. We found that the majority of NeuroD1‐infected astrocytes retained astrocyte markers (GFAP) at 1 wpi (Figure 1C), whereas an increasing number of NeuroD1‐infected astrocytes acquired the neuronal marker NeuN at 3 and 6 wpi (Figure 1D,E, quantified in F). Moreover, a significant number of GFP^+^ cells with both astrocytic and neuronal markers (double‐positive) were detected in the hippocampus of AAV‐NeuroD1 treated mice (Figure 1D,H, and quantified in 1F green bar), whereas these cells were never detected in controls (Figure 1G). At 6 wpi, 53.7% of AAV9‐transduced astrocytes were fully converted into neurons (GFAP^−^/NeuN^+^) by NeuroD1 (Figure 1E and quantified in 1F red bar). Interestingly, we also found that when astrocytes acquired a neuronal marker (NeuN^+^), their GFAP signal was significantly reduced (Figure 1H,I). Of note, the number of double‐positive cells was smaller in the early and late time points after AAV‐NeuroD1 injection, and most in the mid‐stage (3 wpi, Figure 1F green bar), indicating transitional stage cells were observed during AtN conversion (Figure 1H). Together, these data reveal that local AtN conversion has been achieved by ectopic expression of NeuroD1 in 5xFAD mouse hippocampus.

### 2.2 Single Cell RNA‐Sequencing Analysis of AtN Conversion in 5xFAD Mouse Hippocampus

**Figure 2:**
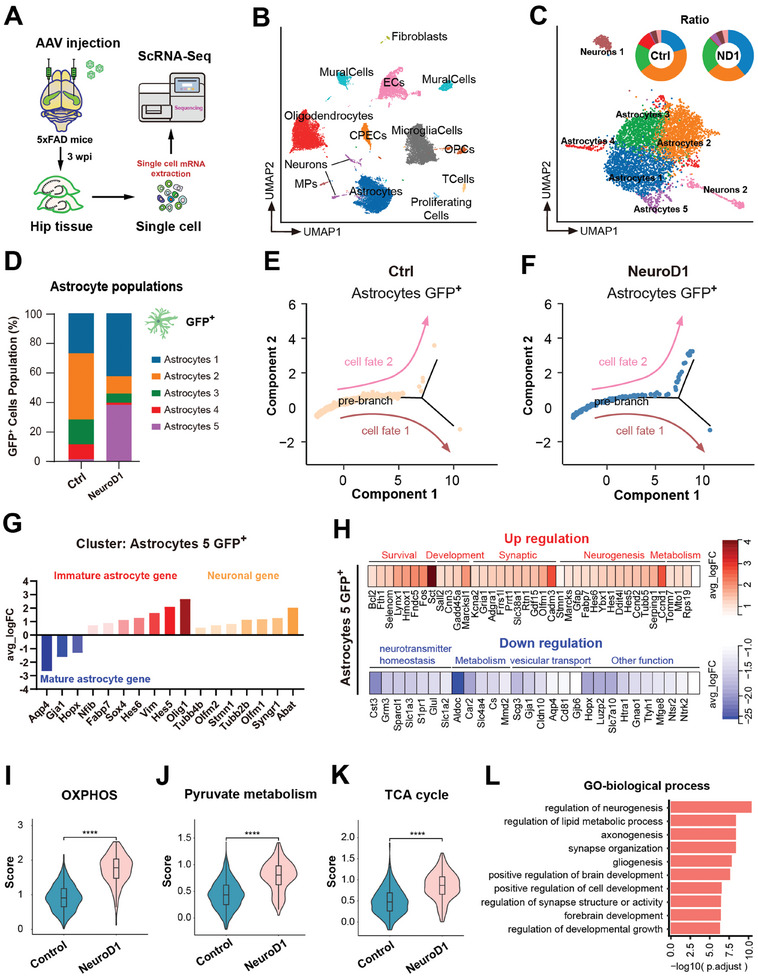
sc‐RNAseq analysis revealed the molecular mechanism of AtN. A) Experimental design. 5xFAD mouse Hippocampi were injected with AAV9 and extracted from control and NeuroD1 treated mice at 3 weeks post injection (*n* = 3 mice per group). Hippocampi were dissociated, and single‐cell suspensions were prepared and isolated using a microfluidic chip, followed by scRNA‐seq processing. B) Clustering using the Seurat package revealed 13 distinct cellular populations. UMAP of 22,752 single‐cell RNA profiles from 5xFAD mouse hippocampi of AAV9 injection. C) UMAP clustering, showing 2 subclusters of neurons, 5 subclusters of astrocytes. Pie chart showing the percentage of different clustered cells in the control and NeuroD1 group. Note that the astrocyte 4 population was present in controls, but the astrocyte 5 population was more abundant in the hippocampus of NeuroD1‐treated mice. D) GFP^+^ astrocyte population in control and NeuroD1 group. Up to 38.2% of GFP^+^ astrocytes with NeuroD1 were clustered in the astrocyte 5 population, but in control GFP^+^ astrocytes this proportion was less than 1.3%. E,F) Pseudotime analysis revealed the difference of GFP^+^ astrocytes in Control (E) and NeuroD1‐GFP group (F). Note that more NeuroD1 overexpressing astrocytes were distributed in the branch toward to neuronal population 2 (cell fate 2, related to Figure S3g, Supporting Information). G) Relative gene expression level of GFP^+^ astrocyte population 5 (NeuroD1 vs Ctrl) in terms of mature astrocyte genes, immature astrocyte genes, and neuronal genes. H) Heatmap showing some of the upregulated and downregulated genes of NeuroD1‐GFP^+^ astrocytes (subpopulation 5) in cell fate 2 direction. I–K) Metabolic pattern score of oxidative phosphorylation (OXPHOS, I), pyruvate metabolism (J), and TCA cycle (K) were increased in NeuroD1 overexpressing astrocytes. L) Gene Ontology terms of the DEGs in response to NeuroD1 overexpression in hippocampal astrocytes.

To elucidate the molecular mechanism of NeuroD1‐mediated AtN conversion in 5xFAD mouse hippocampus, we next performed unbiased single‐cell RNA sequencing (scRNA‐seq) at 3 wpi (**Figure** **2**A), when the greatest number of transition‐state cells (GFAP^+^/NeuN^+^) was detected (Figure 1F). Then, a microwell‐based ultra‐high throughput scRNA‐seq platform was employed in this study. We analyzed scRNA‐seq data of 22 752 cells from the control and NeuroD1‐treated mice and identified 13 clustered cell populations by the marker genes (Figure 2B; Figure S1A, Supporting Information). Astrocytes accounted for ≈24% of total cells, consistent with the expectation. However, most of the neurons were lost because of tissue digestion and dissociation (Figure S1B, Supporting Information left). Among these astrocytes, 1241 and 221 GFP^+^ astrocytes were found in the control and NeuroD1 group, respectively (Figure S1B, Supporting Information right). Next, by analyzing differentially expressed genes (DEGs, fold change > 1.5, *p* < 0.05) in AAV‐infected astrocytes (GFP^+^), we found 85 down‐regulated genes and 151 up‐regulated genes (Figure S1C, Supporting Information). Of note, many neuronal genes (e.g., *Abat, Syn2, Olfm2*, and *Stmn1*) were induced by NeuroD1 overexpression (Figure S1D, Supporting Information), while mature astrocyte genes (e.g., *Slca3, Aqp4, Aldoc*, and *Gja1*) were down‐regulated (Figure S1E, Supporting Information). Moreover, four algorithms (StemID, CytoTrace, SLICE, and CCAT) were employed for estimating cell potency from scRNA‐seq data,^[^ ^18^, ^19^, ^20^, ^21^ ^]^ and showed significantly higher differentiation capacity (entropy value) in NeuroD1 overexpressed astrocytes (Figure S2A–D, Supporting Information). Together, these data reveal that NeuroD1 initiates the AtN conversion, as evidenced by the upregulation of neuronal genes and repression of astrocyte genes.

To further elucidate the characterization of AtN conversion, we separated astrocytes and neurons from the total cell population for further analysis. Neurons were divided into two subpopulations, of which subpopulation 2 neurons highly expressed *Igfbpl1*, *Marcksl1*, and *Tmsb10* genes (Figure S3A, Supporting Information). These genes are mainly related to axon growth, neuronal differentiation, and regeneration, and are mainly expressed in immature neurons.^[^ ^22^, ^23^, ^24^ ^]^ Astrocytes were further divided into 5 subpopulations based on their gene expression profile (Figure 2C; Figure S3A, Supporting Information). Interestingly, in both control and NeuroD1‐treated mice, the majority of astrocytes were clustered in populations 1, 2, and 3, but population 4 astrocytes were primarily present in control mice, whereas subpopulation 5 astrocytes were mainly present in the NeuroD1 group (Figure 2C,D; Figure S3B, Supporting Information). Notably, compared with astrocyte subpopulations of 1–4, mature astrocyte genes were downregulated, while immature astrocyte and neuronal genes were upregulated in subpopulation 5 astrocytes (Figure S3E, Supporting Information). To determine the composition of astrocyte subpopulation 5, we scattered GFP^+^ cells into U‐MAP plots and found that few GFP^+^ cells in the control group were distributed within subpopulation 5 (Figure S3C, Supporting Information), while the majority of NeuroD1 overexpressed GFP^+^ cells are clustered in astrocyte subpopulation 5 (Figure S3D, Supporting Information). Overall, ≈40% of NeuroD1‐overexpressing GFP^+^ cells belonged to subpopulation 5 astrocytes, compared with ≈1% in the control group (Figure 2D, purple block). Next, the reprogramming trajectory of astrocytes was constructed through pseudotime analysis (Figure S3F, Supporting Information), more GFP^+^ astrocytes in the differentiation path from astrocytes to neuronal fate (cell fate 2) were observed in NeuroD1‐treated mouse brain (Figure 2F; Figure S3G, Supporting Information). In addition, cells distributed in the cell fate 2 direction were mainly subpopulation 5 GFP^+^ astrocytes (Figure S3F, Supporting Information), which were abundant in the NeuroD1‐treated hippocampus but very rare in the control group (Figure 2E,F; Figure S3G, Supporting Information). Furthermore, the gene expression profile of group 5 GFP^+^ astrocytes (NeuroD1 vs Ctrl) distributed in the cell fate 2 direction was fundamentally altered, exhibiting a downregulation of mature astrocyte genes (e.g., *Aqp4*, *Gja1*, and *Hopx*), upregulation of immature (such as *Olig1*, *Hes5*, *Vim*, *Hes6* and *Sox4*) and neuronal genes (e.g., *Abat*, *Syngr1*, *Olfm1* and *Tubb4b*; Figure 2G). In addition, overexpression of NeuroD1 in astrocytes (subpopulation 5) also upregulated many genes related to neurogenesis, development, synaptic function, survival, and mitochondrial energy production, while downregulated genes related to neurotransmitter homeostasis, metabolism, vesicle transport, and other functions more commonly observed in astrocytes (Figure 2H). Collectively, our findings demonstrate that NeuroD1 significantly alters the transcriptomic landscape of astrocytes toward neuronal landscape, suggesting a role for NeuroD1 in neuronal fate reprogramming.

Moreover, neurons and astrocytes have distinct energy metabolic profiles. Astrocytes mainly rely on glycolysis (anaerobic) for their energy production, whereas neurons preferentially use oxidative phosphorylation (aerobic) to generate energy from glucose.^[^ ^25^, ^26^ ^]^ As expected, AtN conversion was also accompanied by a change in energy metabolism preference, with a significant elevation of OXPHOS, pyruvate metabolism, and TCA cycle in GFP^+^ cells (Figure 2I–K). Gene Ontology (GO) enrichment analysis revealed that many DEGs were enriched in biological processes including neurogenesis, axonogenesis, synapse organization, and brain development (Figure 2L). Together, these data suggest that transcriptional factor NeuroD1 drives the astrocyte transcriptome in a neuronal direction.

To investigate the specific subtypes of astrocytes targeted for conversion into neurons, we conducted RNA velocity analysis to predict the direction and speed of individual cell transitions between clusters in scRNA‐seq data.^[^ ^27^, ^28^ ^]^ All astrocytes and neurons were included in the unbiased RNA velocity analysis. Interestingly, arrows in astrocyte subpopulation 5 point toward the immature neurons of subpopulation 2 (Figure S4A, Supporting Information), suggesting that cluster 5 astrocytes could be the major source for AtN conversion in 5xFAD mouse hippocampus after NeuroD1 treatment. Subsequently, we analyzed DEGs in A1 (neurotoxic reactive astrocytes) and A2 reactive astrocytes (neuroprotective reactive astrocytes).^[^ ^29^ ^]^ The heatmap analysis demonstrated that 3 A1‐enriched DEGs were downregulated (*C3*, *S1Pr3*, and *Gbp2*), while 1 DEG (*Sperping1*) was upregulated in NeuroD1‐treated astrocytes (Figure S4B, Supporting Information). For A2‐enriched DEGs, we observed 3 upregulated (*Emp1*, *CD109*, and *B3gnt5*) and 1 downregulated (*Cd14*) DEGs in NeuroD1‐treated astrocytes (Figure S4B, Supporting Information). These findings suggest that genes associated with neurotoxic phenotypes are downregulated, whereas those related to neuroprotective phenotypes are upregulated in cluster 5 astrocytes in NeuroD1‐treated mice. Notably, astrocyte‐5 subpopulations were predominantly observed in the brains of NeuroD1‐treated mice (Figure S4C, Supporting Information). Conversely, we noticed that cluster 4 astrocytes were primarily found in the control group (Figure S4C, Supporting Information). Heatmap of the top 10 marker genes used to define cluster 4 astrocytes (Figure S4D, Supporting Information), most of which are involved in promoting inflammatory responses (such as *Cxcl10*, *Ifitm3*, *Serpina3n*, and *Isg15*). Taken together, these data suggest that more neuroprotective reactive astrocytes are generated, whereas proinflammatory reactive astrocytes are suppressed, in the hippocampus of 5xFAD mice treated with NeuroD1.

### 2.3 Brain‐Wide Gene Therapy Targeting Astrocytes for Neuronal Conversion

**Figure 3:**
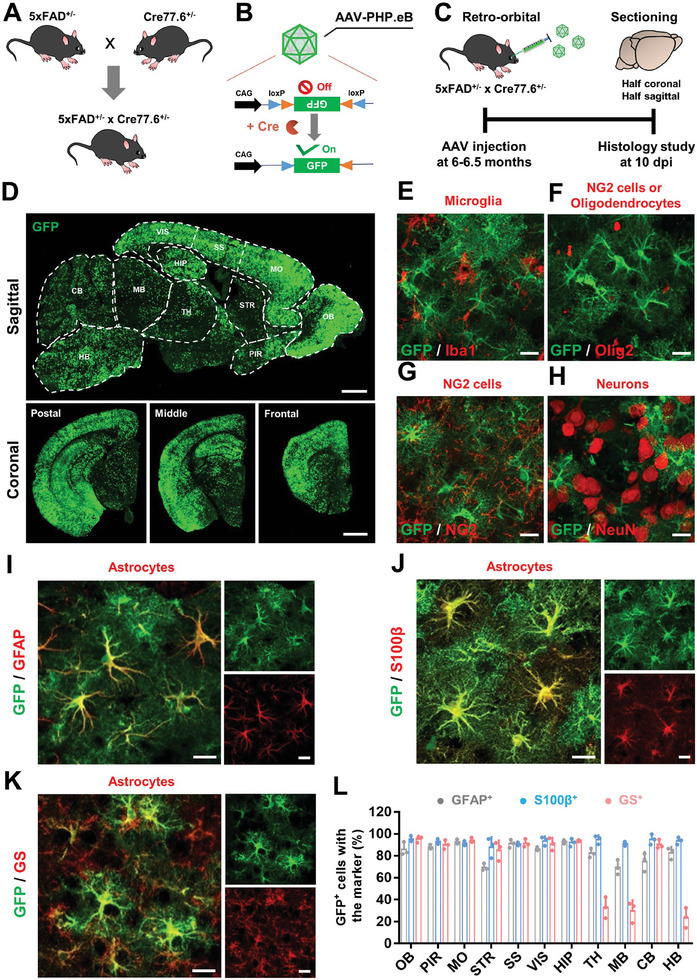
Design and validation of brain‐wide targeting astrocytes for transgene expression in 5xFAD mouse brain. A) Male 5xFAD mice were crossed with *Gfap*::cre transgenic female mice (Cre 77.6) to generate bigenic mice. B) Schematic of the Cre‐FLEx system used to activate GFP expressing and the inverted transgene was packaged into the AAV‐PhP.eB capsids C) Flowchart of study design to evaluate GFP expressing in bigenic mouse brain. dpi, days post injection. D) Representative low‐mag confocal images of GFP expression in the view of sagittal (top, scale bar = 0.5 mm) and coronal (bottom, scale bar = 0.2 mm) sections from anterior to posterior. GFP‐expressing cells were investigated in 11 brain regions as shown in sagittal view. CB, cerebellum; HB, hindbrain; HIP, hippocampus; MB, midbrain; MO, motor cortex; OB, olfactory bulb; SS, somatosensory cortex; STR, striatum; TH, thalamus; VIS, visual cortex; PIR, Piriform cortex. E–H) Typical images showing that GFP was not expressed in microglia (Iba1, E), NG2 cells or oligodendrocytes (Olig2, F), NG2 cells (NG2, G), and Neurons (NeuN, H). Scale bar, 20 µm. I,J) Validation of GFP that was co‐labeled with GFAP (I), S100β (J), and Glutamine synthetase (GS, K). Scale bar, 20 µm. L) Percentage of GFP‐expressing (GFP^+^) cells with three different astrocytic markers in different brain regions (*n* = 3 mice per group, mean ± SD).

The Alzheimer's disease brain is characterized by widespread neurodegeneration, particularly in the hippocampus and cortex, which are two brain regions intimately linked to learning and memory.^[^ ^12^, ^14^ ^]^ To date, we have achieved local AtN conversion in the hippocampus of 5xFAD mice in this study, and in the cortex in our previous study.^[^ ^11^ ^]^ Next, we wondered whether a single AAV‐NeuroD1 treatment could achieve brain‐wide AtN conversion, rather than confined to a localized region, and thus improve cognitive performance in 5xFAD mice. Therefore, we investigated the possibility of achieving brain‐wide astrocyte‐to‐neuron (AtN) conversion in AD mouse brains by using systemic administration of the BBB‐penetrating AAV‐PhP.eB vectors. To trace astrocyte‐converted neurons in the long term, we developed a Cre‐FLEx (flip‐excision) expression system which includes a bigenic mouse line expressing Cre recombinase under the control of the *Gfap* promoter (Cre77.6) to target astrocytes in 5xFAD mouse brains. AAV‐PhP.eB FLEx vectors with an inverted coding sequence of GFP (control) or NeuroD1‐P2A‐GFP driven by a strong universal synthetic promoter CAG were employed to trace the cell fate of Cre‐expressing astrocytes in 5xFAD brains after overexpressing GFP or NeuroD1‐GFP (**Figure** **3**A–C). As expected, we observed a wide distribution of GFP across the whole brain including the cerebral cortex and hippocampus (Figure 3D), suggesting that systemic delivery of AAV‐PhP.eB‐CAG::FLEx‐GFP in bigenic Cre‐AD mice achieved brain‐wide expression of the ectopic gene.

To identify the cell type of GFP‐expressing cells, we immunostained several brain residential cell markers and found that GFP was rarely expressed in microglia (Iba1^+^), oligodendrocytes (Olig2^+^), NG2 cells (NG2^+^), or neurons (NeuN^+^, Figure 3E–H). On the other hand, over 80% GFP^+^ cells were co‐expressed with astrocytic marker GFAP in various brain regions (Figure 3I,L and Figure S5, Supporting Information). Almost all GFP^+^ cells were co‐labeled with astrocyte pan marker S100β across the different brain areas (Figure 3J, quantified in Figure 3L). Interestingly, in most regions of the mouse brain, more than 90% of GFP^+^ cells were labeled by glutamine synthetase (GS), another commonly used astrocyte marker, but we also observed that more than half of the GFP^+^ cells located in the thalamus, midbrain, and hindbrain did not co‐express GS (Figure 3K,L). These results might be attributed to the fact that oligodendrocytes in those brain regions also express GS.^[^ ^30^ ^]^ Together, these data suggest that systemic delivery of Cre‐dependent AAV‐PhP.eB‐CAG::FLEx‐GFP in bigenic Cre‐AD mice enables brain‐wide targeting of astrocytes.

### 2.4 NeuroD1‐Mediated Neuronal Conversion among Different Brain Regions

**Figure 4:**
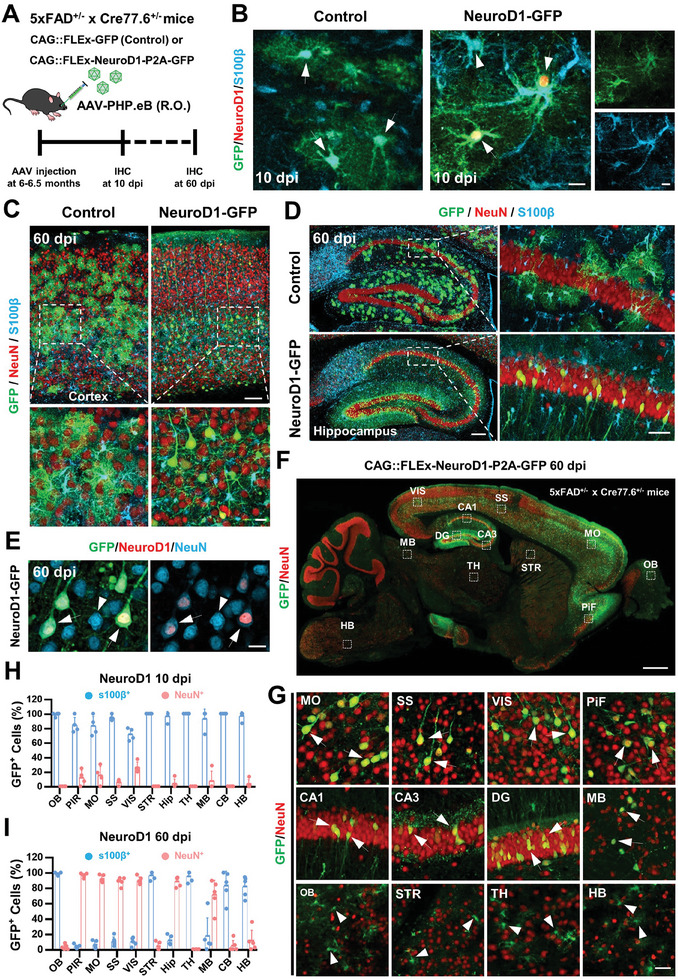
Brain‐wide AtN conversion study in 5xFAD mouse brain. A) Study design to examine NeuroD1‐mediated AtN conversion. B) Representative confocal image showing that NeuroD1 was expressed in AAV‐infected astrocytes (right, GFP^+^ and S100β^+^, arrows), but not in non‐infected astrocytes (right, GFP^−^ and S100β^+^, arrowhead) and control AAV‐infected astrocytes (left, GFP^+^ and S100β^+^, arrows) at 10 dpi. Scale bar, 20 µm. C) Low‐mag confocal images of cortical areas at 60 dpi. GFP^+^ cells were co‐labeled with astrocytic marker S100β in the control mouse brain (left), but in the NeuroD1‐treated mouse brain, they were co‐labeled with the neuronal marker NeuN (right). Scale bar, 100 µm (top), 20 µm (bottom). D) Typical images showing NeuroD1‐mediated AtN conversion in bigenic mouse hippocampus. Scale bar, 200 µm (left), 50 µm (right). E) NeuroD1 immunostaining signals were detected within GFP^+^ neurons (arrows) but not in the endogenous neurons (arrowhead) at 60 dpi. Scale bar, 20 µm. F) A sagittal view of 5xFAD mouse brain at 2 months after AAV‐PhP.eB‐NeuroD1‐P2A‐GFP injection (R.O.). Scale bar, 200 µm. G) Enlarged confocal images that were indicated by the white dashed box in panel F, Scale bar, 20 µm. H,I) Percentage of GFP^+^ cells with S100β and NeuN co‐expression in mouse brains that were treated with NeuroD1 at 10 dpi *(n* = 4 mice, H) and 60 dpi (*n* = 5 mice, I), respectively. Note that NeuroD1‐mediated AtN conversion efficiency differs obviously in different brain regions. Data was shown as mean ± SD.

Astrocytes are known to have region‐specificity.^[^ ^31^, ^32^ ^]^ Whether NeuroD1‐mediated AtN conversion can be achieved in different brain regions is largely unknown. To address this topic, AAV‐PhP.eB‐CAG::FLEx‐NeuroD1‐P2A‐GFP was injected retro‐orbitally (R.O.) into Cre‐AD bigenic mice and analyzed at 10 days post injection (dpi) and 60 dpi for immunohistochemistry study (**Figure** **4**A). At an early time‐point (10 dpi), we observed a strong NeuroD1 signal in S100β^+^ astrocytes but not in control (Figure 4B), indicating that AAV‐infected astrocytes began to express conversion factor NeuroD1. By 60 dpi, many GFP‐expressing cells in control mice still showed astrocytic morphology and were labeled by pan astrocyte marker S100β (Figure 4C,D top). However, NeuroD1‐overexpressed GFP^+^ cells exhibited typical neuronal morphology with NeuN immunostaining signal throughout the cortex (Figure 4C right) and hippocampus (Figure 4D bottom). Furthermore, NeuroD1 immunostaining signals were detected in GFP^+^ neurons at 60 dpi (Figure 4E), whereas NeuroD1 was only observed in astrocytes at 10 dpi (Figure 4B right). Therefore, these results indicate that overexpression of NeuroD1 in astrocytes induces AtN conversion in the cortex and hippocampus in Alzheimer's mouse brains.

To further study the differences in NeuroD1‐mediated AtN conversion efficiency in different brain regions, we analyzed 11 regions on sagittal sections of mouse brains (Figure 4F). At 60 dpi, we found many GFP^+^ neurons (NeuN^+^) located in the cortex (Figure 4G top), hippocampus, and parts of the midbrain (Figure 4G middle row right), but GFP^+^ cells in the olfactory bulb, striatum, thalamus, and hindbrain were not co‐stained with NeuN (Figure 4G bottom). Quantitative analyses showed that the majority of GFP^+^ cells at 10 dpi after AAV‐NeuroD1 treatment were still astrocytes (S100β^+^), but after 60 dpi of AAV‐NeuroD1 treatment, more than 80% of GFP^+^ cells expressed neuronal marker (NeuN), and these cells were widely distributed in the cerebral cortex, hippocampus and the midbrain area (Figure 4H,I). However, in other brain regions, most GFP^+^ cells were still co‐labeled with astrocyte marker (S100β) after 60 days of NeuroD1 treatment (Figure 4I). These data suggest that region‐specific astrocytes exhibit heterogeneous susceptibility to NeuroD1‐mediated AtN conversion.

Next, we wondered whether AtN conversion in the cortex and hippocampus could affect Aβ pathology and reactive gliosis in 5xFAD mice and therefore performed Aβ42 and GFAP immunostaining. Then, we found there is no significant difference in Aβ42 between control and NeuroD1 groups, suggesting Aβ42 pathology is not affected by AtN conversion in 5xFAD mouse brain (Figure S6A,B, Supporting Information). Interestingly, despite there is no significant difference in GFAP immunostaining in the cortex between these two groups, more hypertrophic reactive astrocytes were observed in the hippocampus of 5xFAD mice in the control group (Figure S6C,D, Supporting Information). Thus, these results suggest that neurotoxic reactive astrocytes in the hippocampus of 5xFAD mice are decreased by NeuroD1‐mediated AtN conversion.

### 2.5 Brain‐Wide Gene Therapy Conversion Regenerated Half a Million New Neurons in 5xFAD Mouse Brain

**Figure 5:**
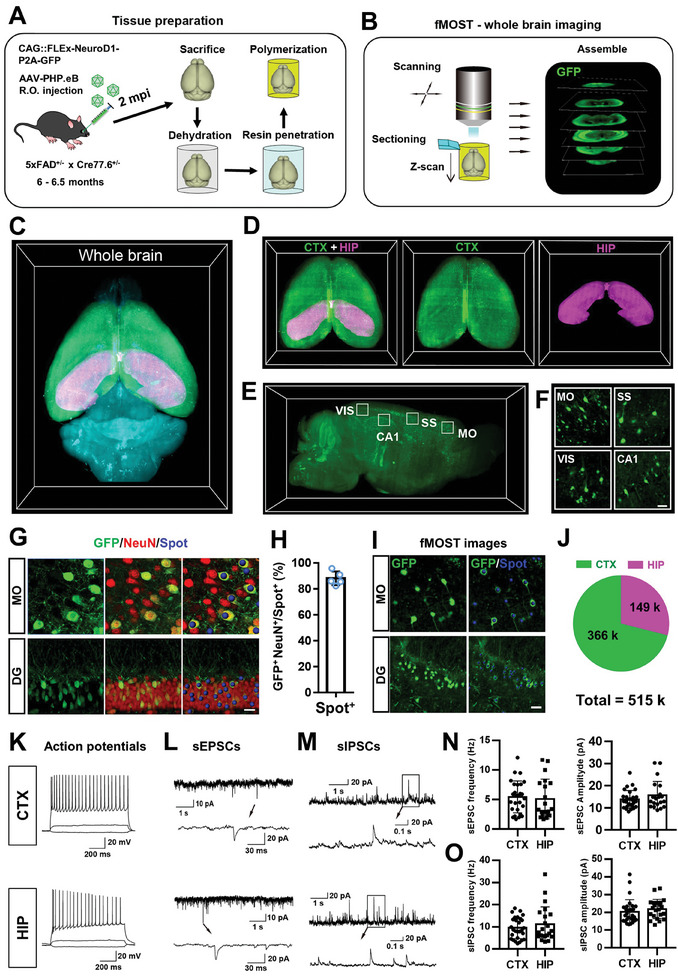
AtN whole brain imaging and electrophysiologic study. A) Schematic diagram showing the collection and preparation of mouse brain tissue for fMOST imaging. B) Workflow of fMOST whole brain imaging procedure, the resin‐embedded brain tissues were cut by the diamond knife in an anteroposterior direction, with 2 µm thickness step by step. C) Representative images of whole‐brain GFP fluorescent imaging by fMOST. Green, purple and cyan represent the cortex, hippocampus, and cerebellum, respectively. D) Imaris software was used to extract the cortical (green) and hippocampal (purple) structures from the whole mouse brain for further independent analysis. E) A 3‐D transparent view from the sagittal plane (100 µm thickness), the white box indicated some typical brain regions in the cortex (MO, SS, and VIS) and hippocampus (CA1). MO, motor cortex; SS, somatosensory cortex; VIS, visual cortex; CA1, cornu ammonis 1. F) Representative high‐magnification images from single‐layer fMOST fluorescence images (GFP). Scale bar, 20 µm. G) Use confocal images to test the accuracy of Imaris software in identifying converted neurons (GFP^+^ and NeuN^+^), the blue spots represent converted neurons defined by the Imaris software. Scale bar, 20 µm. H) The statistical data shows that the accuracy of converted neuron identification by Imaris software reaches ≈90%. I) Examples of Imaris software automatically identify astrocyte‐converted neurons (GFP^+^ and indicated by blue spots) in fMOST fluorescence images. Scale bar, 20 µm. J) Quantitative results showed that NeuroD1 induced 36.6 and 14.9 k astrocyte‐converted neurons in the cortex and hippocampus, respectively (*n* = 4 mice). K–M) Representative traces of evoked repetitive action potentials (K), spontaneous excitatory post‐synaptic currents (sEPSCs, L), and inhibitory post‐synaptic currents (sIPSC, M) in converted neurons located in the cortex (top) and hippocampus (bottom). N) Summary graph of mean frequency and amplitude of sEPSCs. O) Bar graph of mean frequency and amplitude of sIPSCs. Data was shown as mean ± SD.

We next asked how many neurons that we can regenerate in the 5xFAD mouse brain by brain‐wide AtN conversion. To achieve this goal, the fluorescence micro‐optical sectioning tomography (fMOST) technique was employed in this study (**Figure** **5**A,B), a technique developed to obtain high‐resolution images of samples labeled with fluorescent dyes and recently were mainly used to get the whole‐brain image with single‐neuron resolution. Whole‐brain GFP imaging was then achieved using fMOST on the bigenic mice 60 days after NeuroD1 treatment (Figure 5C–F, *n* = 4 mice). Since there are huge differences in morphology between neurons and astrocytes, we can easily identify the converted neurons (GFP^+^ and NeuN^+^) using the spot function of Imaris software. As shown in the confocal images (Figure 5G), converted neurons (GFP^+^ and NeuN^+^) were efficiently labeled by the blue spots with high accuracy (≈90%, Figure 5H), which enabling us to count GFP^+^ converted neurons in the whole mouse brain. Next, fMOST images were loaded into Imaris software for quantification of converted neurons, and the converted neurons were automatically identified and labeled with the blue spots (Figure 5I). We found that NeuroD1‐mediated brain‐wide gene therapy conversion regenerated ≈366 000 new neurons in the cortex and 149 000 new neurons in the hippocampus, respectively (Figure 5J). These results indicate that whole‐brain gene therapy conversion regenerated a total of >500 000 new neurons in a 5xFAD mouse brain.

We further assessed the electrophysiological functions of astrocyte‐converted neurons induced by NeuroD1 in the cortex and hippocampus of 5xFAD bigenic mice. Whole‐cell recordings were performed on acute cortical and hippocampal brain slices at 60–90 dpi following injection of NeuroD1 viral vectors. Repetitive action potentials were induced upon injecting depolarization currents in both cortical (Figure 5K top) and hippocampal converted neurons (Figure 5K bottom). Furthermore, we recorded significant spontaneous synaptic currents, both excitatory and inhibitory, in all converted neurons (Figure 5L–O), indicating that these neurons had formed synaptic connections with other neurons and therefore integrated into functional neural circuits.

### 2.6 Converted Neurons Acquire Local Neuronal Identities

**Figure 6:**
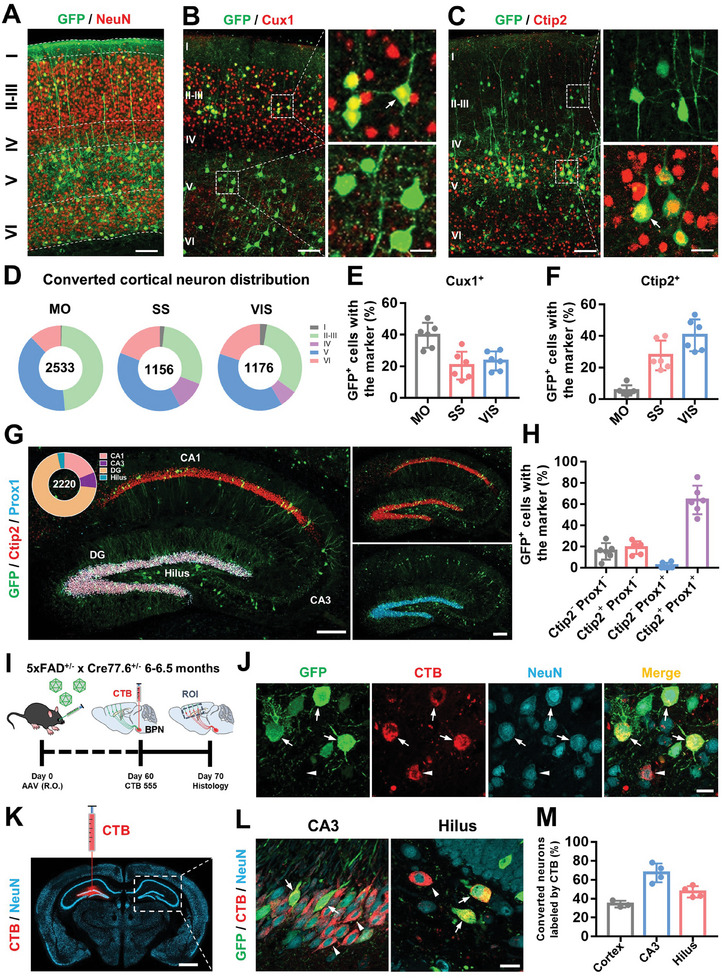
Brain‐wide astrocyte‐converted neurons acquire adequate position identity and axonal projection. A) Representative confocal images showing the spatial distribution of the converted neurons (GFP^+^) in the mouse cortex at 60 dpi of AAV‐PhP.eB‐NeuroD1. White dashed lines indicate the laminar boundaries within the bigenic mouse cortex. Scale bar, 100 µm. B) Example images show that CUX1‐expressing converted neurons (CUX1^+^ and GFP^+^) are predominantly located in superficial layers (II‐III), whereas converted neurons in deeper layers (V‐VI) lack CUX1 expression. White dashed boxes were enlarged in the right panels and the white arrow indicated a typical converted neuron that expressing CUX1. Left scale bar, 100 µm; right scale bar, 20 µm. C) Astrocyte‐converted neurons were marked by Ctip2, a deep layer marker of cortex (V‐VI). Note that converted neurons located in shallower layers did not express Ctip2. The white dashed box indicates the magnified area, and the white arrow indicates the Ctip2‐expressing converted neurons. Left scale bar, 100 µm; right scale bar, 20 µm. D) Pie chart illustrating the distribution of astrocyte‐converted neurons in subregions of the cortex. The number in the middle of the pie chart represents the total number of converted neurons (*n* = 6 mice). MO: motor cortex, SS: somatosensory cortex, VIS: visual cortex. E,F) Quantifications of Cux1^+^ (E) and Ctip2^+^ (F) converted neurons in different subregions. G) Distribution of astrocyte‐converted neurons in bigenic mouse hippocampus. Note that Ctip2 was highly expressed in CA1 and DG but not in CA3 and hilus, whereas prox1 was only expressed in DG. Pie chart (top left) indicating the subregion distribution of converted neurons (*n* = 2220 neurons from 6 mice). Scale bar, 200 µm. H) Bar graph showing the percentage of the converted neurons with different identity markers. I) Study design of CTB retrograde tracing of converted neurons in bigenic mouse cortex. ROI: region of interest. J) Confocal images showing CTB retrograde labeled converted neurons (arrows) and pre‐existing neurons (arrowhead) in cortical ROI. Scale bar, 20 µm. K) A typical image showing CTB was injected in one side of the hippocampus, and the contralateral side of the hippocampus was investigated. Scale bar, 1 mm. L) Representative confocal images showing CTB‐labeled converted neurons (arrows) and pre‐existing neurons (arrowheads) in CA3 and hilus, respectively. Scale bar, 20 µm. M) Quantification data showing CTB labeled converted neurons in the cortex (*n* = 3 mice), CA3 (*n* = 4 mice), and hilus (*n* = 4 mice).

The brain is a highly structured organ, with specific types of neurons in particular locations forming a complex neural network. To examine the subtypes of the converted neurons and their spatial distribution, we focused on laminar and molecular properties. In the cerebral cortex, there was a widespread presence of converted neurons throughout the upper and lower layers (**Figure** **6**A). Most Cux1^+^ converted neurons were localized to the upper layers (Figure 6B, GFP^+^), while Ctip2^+^ converted neurons mainly resided in deeper layers (Figure 6C, GFP^+^), similar to the spatial distribution of endogenous neurons (GFP^−^). The converted neurons were mainly distributed in layers II‐III and V‐VI, with a small portion in layer IV and a few in layer I (Figure 6D). Of these converted neurons, the majority of Cux1^+^ superficial layer neurons were located in the motor cortex, while over 90% Ctip2^+^ deep layer neurons were regenerated in the somatosensory and visual cortex (Figure 6E,F). In the hippocampus, the presence of converted neurons can be detected in different regions including CA1, CA3, and dentate gyrus (DG). Among them, ≈70% of the converted neurons were located in the granule cell layer of DG (Pie chart in Figure 6G). Specifically, we observed that converted neurons in the CA1 region only expressed Ctip2, and the granular layer of the dentate gyrus (DG) co‐expressed Ctip2 and Prox1, whereas converted neurons in the CA3 and hilus regions did not express Ctip2 or Prox1 (Figure 6G). Among them, NeuroD1‐mediated AtN conversion regenerated a large proportion of Ctip2 and Prox1 double positive converted neurons in DG, but very few Prox1^+^ converted neurons in the hippocampus (Figure 6H). Together, these data indicate that astrocyte‐converted neurons adopt the molecular signatures characteristic of local neuronal subtypes with spatial locations in the cerebral cortex and hippocampus.

Since all of the antibodies we used above labeled glutamatergic neurons, we next asked whether NeuroD1‐mediated astrocyte‐to‐neuron conversion could regenerate GABAergic neurons in the 5xFAD mouse brain. Surprisingly, few of the converted neurons were co‐labeled with the GABAergic neuronal marker GAD67 throughout the entire cortex and hippocampus (*n* = 6 mice, Figure S7, Supporting Information). These data suggest that FLEx‐CAG::NeuroD1 is unable to convert cortical and hippocampal astrocytes into GABAergic neurons in the brains of Cre‐AD mice. Since most of the converted neurons are glutamatergic, it is unclear whether this would result in an imbalance in excitatory–inhibitory (E–I) activity. Therefore, we conducted patch‐clamp recordings on hippocampal endogenous neurons in 5XFAD brains with (NeuroD1) and without (Ctrl) AtN conversion. No significant differences in sEPSC and sIPSC were found between groups (Figure S8, Supporting Information), indicating that NeuroD1‐regenerated glutamatergic neurons did not disrupt the E–I balance in the hippocampus of 5xFAD mice.

Having shown that NeuroD1‐converted neurons acquired proper neuronal subtypes and spatial location identities, we next examined whether these newly generated neurons could project their axons to the appropriate brain regions. The corticopontine tract is a distinctly convergent descending motor pathway that contributes more than 95% of afferents to the basal pontine nucleus (BPN).^[^ ^33^ ^]^ Therefore, this pathway is particularly advantageous for studying ipsilateral axonal projections of converted neurons located in the deeper layer of the cortex. A retrograde tracer cholera toxin subunit B (CTB) was injected into the BPN at 60 days after injection of AAV‐PhP.eB‐NeuroD1 (R.O.). Mouse brains were collected 10 days after CTB injection and sagittally sectioned for histological study (Figure 6I). Injection of CTB into BPN labeled 40% of the deep layer converted neurons in the cortex (Figure 6J,M). Next, we focused on the bilateral hippocampus for examining contralateral axonal projections, given the substantial number of neural fibers connecting these structures across both hemispheres. We performed a unilateral injection of CTB into one side of the hippocampus at 60 dpi of AAV‐PhP.eB‐NeuroD1 injection, followed by an analysis of CTB retrograde‐labeled neurons on the contralateral side of the hippocampus 10 days after CTB administration (Figure 6K). About 60% and 50% of the converted neurons were found in CA3 and hilus areas contralaterally to the injection side, respectively (Figure 6L,M). Therefore, these data demonstrate that astrocyte‐converted neurons can extend their axonal projections to the appropriate target brain areas.

### 2.7 Brain‐Wide AtN Conversion Improves Cognitive Performance in 5xFAD Mice

**Figure 7:**
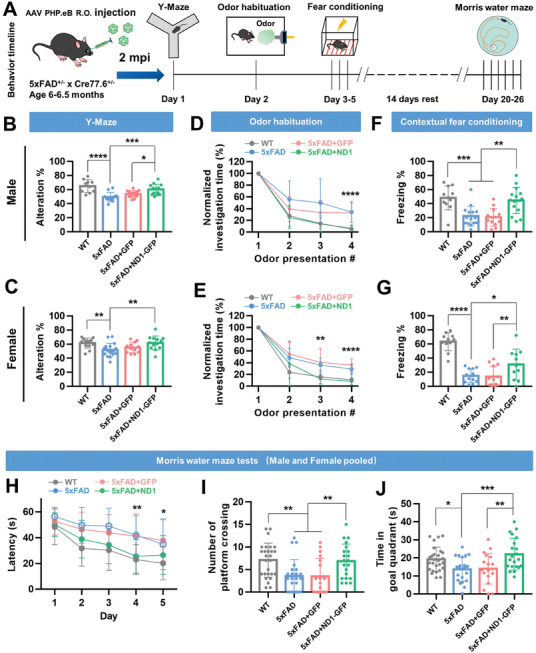
Brain‐wide AtN gene therapy rescues cognition in 5xFAD mice. A) Timeline of behavior experiments. Bigenic mice were received AAV‐PhP.eB (R.O.) at the age of 6–6.5 months. Behavior tests were performed at 2‐months post injection (mpi) of AAV‐PhP.eB. B,C) Y‐maze testing showed that spatial working memory in the spontaneous‐alteration Y‐maze test was significantly impaired in both male (*n* = 11, B) and female (*n* = 18, C) 5xFAD mice compared with WT mice (male, *n* = 10; female, *n* = 13). Systemic delivery of AAV‐PhP.eB‐mediated NeuroD1 improved spontaneous alternation behavior in male (*n* = 13, B) but not in female (*n* = 13, C) 5xFAD mice compared with the GFP control group (male, *n* = 14; female, *n* = 12). *p < 0.05, **p < 0.01, ***p < 0.001, ****p < 0.0001, one‐way ANOVA with Fisher's LSD test. D,E) Normalized investigation time of mice to repeated presentations of the same odor. Olfactory memory was remarkably improved in both male (WT, *n *= 10; 5xFAD, *n* = 9; 5xFAD + GFP, *n* = 13; 5xFAD + ND1, *n* = 14, D) and female (WT, *n *= 9; 5xFAD, *n* = 13; 5xFAD + GFP, *n* = 10; 5xFAD + ND1, *n* = 10, E) 5xFAD mice after NeuroD1 treatment. **p < 0.01, ****p < 0.0001, one‐way ANOVA with Tukey HSD test. F,G) Both male (*n* = 11, F) and female (*n* = 15, G) 5xFAD mice exhibit contexture fear conditioning memory impairment, but NeuroD1‐mediated brain‐wide AtN gene therapy significantly improved fear memory in both male (*n* = 13, F) and female (*n* = 15, G) 5xFAD mice. *p < 0.05, **p < 0.01, ***p < 0.001, ****p < 0.0001, one‐way ANOVA with Tukey HSD test for F and Fisher's LSD test for G. H) Latency to reach the hidden platform during spatial training in the Morris water maze. Compared to WT mice (*n *= 17), 5xFAD mice (*n* = 17) spent a longer time to find the hidden platform (*p* < 0.02, one‐way ANOVA with Fisher's LSD test). Note that 5xFAD mice treated with NeuroD1 (*n* = 21) took less time to find the hidden platform compared to the control group (*n* = 18). *p < 0.05, **p < 0.01, one‐way ANOVA with Fisher's LSD test. I) Number of platform crossings during probe trial (WT, *n* = 17; 5xFAD*, n* = 17; 5xFAD + GFP, *n* = 18; 5xFAD + ND1, *n *= 21. **p < 0.01, one‐way ANOVA with Fisher's LSD test). J) Time spent in the goal quadrant during the probe trial (WT, *n* = 17; 5xFAD, *n* = 17; 5xFAD + GFP, *n* = 18; 5xFAD + ND1, *n *= 21. *p < 0.05, **p < 0.01, ***p < 0.001, one‐way ANOVA with Fisher's LSD test). Data as shown mean ± SD.

We have shown that the systemic delivery of NeuroD1 globally converts astrocytes into neurons, specifically in the cortex and hippocampus, two brain regions closely associated with cognitive function. Then, we assessed its potential effects on the cognitive performances of 5xFAD mice by using four different behavioral tests, including the Y‐maze spontaneous alternation test, odor habituation test, contextual fear conditioning test, and Morris water maze test (**Figure** **7**A).

The Y‐maze spontaneous alternation test is used to evaluate short‐term memory in mice. Compared to the wild‐type (WT) mice (gray bar), both female and male 5xFAD mice exhibited a significantly lower percentage of spontaneous alternation across all entries (Figure 7B,C; blue bar), whereas no significant differences were found in terms of total entries (Figure S9, Supporting Information). Notably, male 5xFAD mice that received NeuroD1 brain‐wide gene therapy exhibited a significantly higher percentage of spontaneous alternation than those not treated with NeuroD1 (Figure 7B, green bar). However, this beneficial result was not observed in the female 5xFAD mice (Figure 7C). This data suggests that the short‐term memory of male 5xFAD mice is enhanced by NeuroD1‐mediated AtN conversion gene therapy.

Olfactory impairment has been recognized as a potential preclinical marker for Alzheimer's disease (AD), and this deficiency has been replicated in animal models of AD.^[^ ^16^ ^]^ The establishment of mouse olfactory habituation tests relies on the basis of spontaneous drinking or foraging, innate odor memory, and curiosity about unfamiliar odors. Typically, WT mice would quickly lose interest on the same odor, shown by decreased time investigating the same odor (Figure 7D,E, gray line). In contrast, 5xFAD mice spent much more time on exploring the familiar odors than the WT mice (Figure 7D,E, blue line). On the other hand, both female and male 5xFAD mice treated with NeuroD1 spent significantly less time on the familiar odors compared to those without NeuroD1 treatment (Figure 7D,E, green vs red line). These results indicate that the impairment of olfactory memory of 5xFAD mice can be rescued by NeuroD1‐mediated gene therapy.

The contextual fear conditioning memory test is a procedure used to assess the strength of the fear memory in a specific context, such as a particular chamber. Mice were placed into the conditioning chamber 24 h after foot‐shock, both female and male 5xFAD exhibited less freezing time in the conditioning chamber compared to the WT mice, suggesting impaired memory. 5xFAD mice in the control group continued to show reduced freezing levels, as expected (Figure 7F,G). In contrast, the freezing time of the NeuroD1‐treated mice recovered to the levels of the WT group, and was significantly higher than the 5xFAD mice without receiving the NeuroD1 (Figure 7F,G), suggesting a rescue of the fear memory deficit by brain‐wide NeuroD1 gene therapy.

The Morris water maze task is one of the most classic behavior assays for assessing spatial learning and memory abilities in AD mice. As expected, 5xFAD mice showed severe deficits in spatial memory formation in comparison to WT mice (Figure 7H). Interestingly, 5xFAD mice receiving NeuroD1 brain‐wide gene therapy were significantly rescued from spatial memory impairment compared with control 5xFAD mice (Figure 7H). In the probe trial, WT mice showed more platform crossing frequency and time in the goal quadrant than 5xFAD mice, as expected (Figure 7I,J). Again, NeuroD1‐treated 5xFAD mice returned to the levels of the WT group, which exhibited higher crossing frequency and more time in the goal quadrant than 5xFAD mice in the control group (Figure 7I,J). These data collectively demonstrate that NeuroD1‐mediated brain‐wide gene therapy leads to a significant enhancement of spatial learning and memory in 5xFAD mice.

### 2.8 Astrocyte‐Converted Neurons are Necessary for Improvement of Fear Memory in 5xFAD Mice

**Figure 8:**
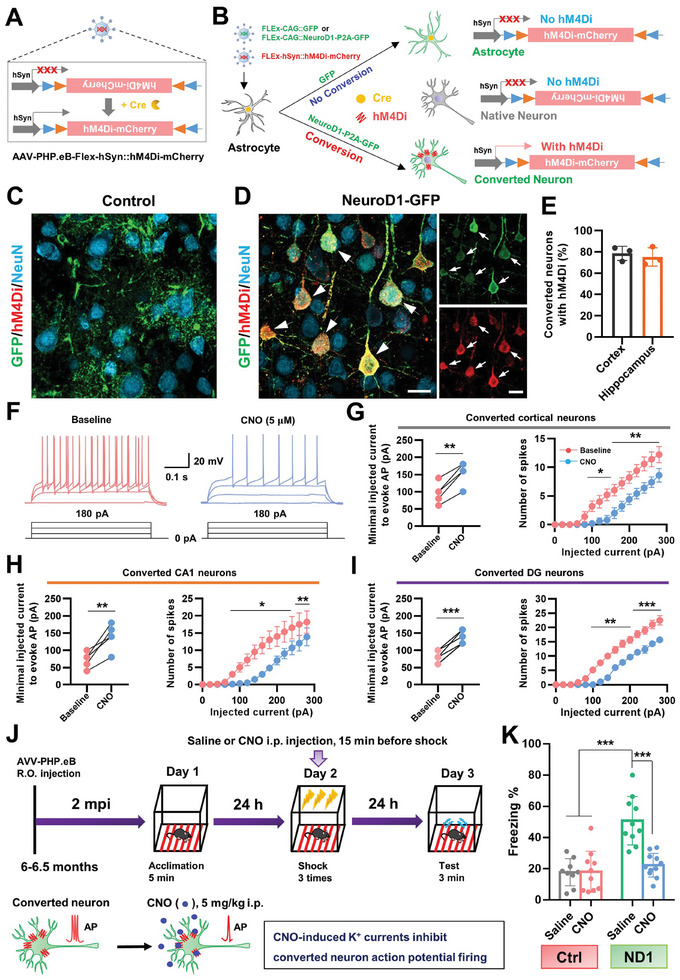
Chemogenetic evidence that astrocyte‐converted neurons contribute to memory improvement in 5xFAD mice. A) Work model of hM4Di‐mCherry AAV‐PhP.eB employing the Cre‐on strategy. The hSyn promoter was used to drive the expression of hM4Di specifically in neurons. B) Systemic injection (R.O.) mixture of AAV‐PhP.eB‐FLEx‐CAG::GFP and FLEx‐hSyn::hM4Di‐mCherry or AAV‐PhP.eB‐FLEx‐CAG::NeuroD1‐P2A‐GFAP and FLEx‐hSyn::hM4Di‐mCherry into the bigenic mice. Due to the dual control of the Cre‐on and the hSyn promoter, only astrocyte‐converted neurons could express hM4Di. C) Typical confocal image showing that hM4Di was not expressed in astrocyte and pre‐existing neurons in the control group at 2‐months post AAV injection. Scale bar, 20 µm. D) Representative image showing that hM4Di was specifically expressed in the converted neurons (GFP^+^, arrows) but not in the pre‐existing neurons at 2‐months post AAV injection. Scale bar, 20 µm. E) Quantified data of astrocyte‐converted neurons with hM4Di. F) Representative evoked action potential traces of astrocyte‐converted neurons before and during CNO perfusion (5 µm). G–I) Left, minimal injected current to induce action potentials (APs) was increased by CNO (5 µm) application in cortical (G, *n* = 5), CA1 (H, *n* = 6), and DG‐converted neurons (I, *n* = 6). Right, a number of induced APs at different injecting currents in cortical (G, *n* = 5), CA1 (H, *n* = 6), and DG‐converted neurons (I, *n* = 6). *p < 0.05, **p < 0.01, ***p < 0.001, Two‐sided paired *t‐*test. J) Schematic (top) and mechanism (bottom) of chemogenetic experimental design demonstrating that astrocyte‐converted neurons directly contribute to the improvement of fear‐conditioned memory in 5xFAD mice 2 months after AAV‐PhP.eB injection. K) Quantification of percentage freezing time of control group mice and NeuroD1 treated mice that were injected (i.p.) with saline or CNO (5 mg kg^−1^). ***p < 0.001, one‐way ANOVA with Tukey HSD test. Data as shown mean ± SD.

Because cognitive functions were remarkably improved by brain‐wide AtN conversion gene therapy, we next examined whether the astrocyte‐converted neurons made a significant contribution to cognition enhancement in 5xFAD mice. To achieve this, cre‐dependent expression of hM4Di, an inhibitory designer receptor exclusively activated by designer drugs (DREADD) technology was employed. We first mixed FLEx‐hSyn::hM4Di‐mCherry and FLEx‐CAG::NeuroD1‐P2A‐GFP/GFP (control) vectors, then injected the AAV mixture into the bigenic mice. Therefore, the FLEx vector is expected to be activated in astrocytes in mice with the *Gfap*‐cre background, which ensures that hM4Di is not expressed in native neurons. In addition, due to the presence of the neuron‐specific promoter hSyn, hSyn::hM4Di should not be expressed in astrocytes, but can be expressed in neurons that are converted from astrocytes (**Figure** **8**A,B). As expected, immunohistochemical analysis confirmed that hM4Di was expressed neither in astrocytes nor in native neurons in the control mouse brain (Figure 8C). Consistently, we observed many converted neurons that were labeled by GFP in NeuroD1‐treated mouse brains (Figure 8D). Moreover, we also detected many hM4Di‐immunoreactive signals that were exclusively expressed in converted neurons but not in native neurons (Figure 8D). Quantified data revealed that ≈80% of astrocyte‐converted neurons expressed hM4Di in the cortex and hippocampus (Figure 8E). Electrophysiological analysis of acute brain slices confirmed that clozapine‐N‐oxide (CNO, 5 µm) inhibited spiking in hM4Di‐expressing converted neurons (Figure 8F), resulting in an elevated spiking threshold and reduced spiking number under the current step injection in converted neurons from cortex (Figure 8G), CA1 (Figure 8H) and DG (Figure 8I), respectively. Taken together, these results indicate that the electrophysiological activity of astrocyte‐converted neurons can be selectively inhibited by DREADD.

We next investigated whether cognition enhancement in 5xFAD mice was due to the brain‐wide AtN conversion gene therapy. Male bigenic mice (6 months) were injected (R.O.) with AAV‐PhP.eB‐NeuroD1, then contextual fear conditioning memory tests were performed at 2 months after AAV‐PhP.eB‐NeuroD1 injection. To inhibit the activity of astrocyte‐converted neurons during a stimulus association period, we injected CNO (5 mg kg^−1^, i.p.) 15 min before the electric shock, the same volume of saline was given as the vehicle control. After 24 h, mice were tested for freezing behavior in the same contexture box (Figure 8J). In the control group (GFP + hM4Di), mice exhibited no difference in freezing behavior on the test day (day 3) between CNO and saline injections, indicating that CNO itself did not have a significant effect on contextual fear memory in 5xFAD mice (Figure 8K). Interestingly, NeuroD1‐treated mice displayed significantly enhanced freezing behavior when they had received a saline injection, whereas this improvement in fear memory was inhibited by CNO injection (Figure 8K). Notably, when NeuroD1‐treated mice were injected with CNO, they displayed similar levels of freezing behavior as control mice (Figure 8K). Therefore, these results indicate that astrocyte‐converted neurons contribute to the improvement of contextual fear memory in 5xFAD mice.

## 3 Discussion

Neuron replacement therapy has long been a hope for patients with Alzheimer's disease. Here, we demonstrated for the first time that brain‐wide AtN conversion gene therapy can significantly improve cognitive function in 5xFAD mice, a commonly used Alzheimer's disease mouse model with severe neuronal loss.^[^ ^34^ ^]^ By using a BBB‐crossing AAV‐PhP.eB viral vectors, systemic administration of this NeuroD1‐mediating AtN system in transgenic mice achieves remarkable neuroregeneration in multiple brain regions, especially in cortex and hippocampus, which are highly related to learning and memory. The converted neurons could stay at the right position and form long‐distance axonal projection. Most importantly, behavior tests show a comprehensive cognitive promotion in 5xFAD mice after brain‐wide AtN gene therapy. Using hM4Di‐DREADD‐mediated chemogenetic inhibition to specifically inhibit the firing probability of converted neurons, precludes cognitive enhancement by brain‐wide AtN gene therapy. Overall, systemic AAV injection targeting resident astrocytes inside the brain for AtN resulted in efficient brain‐wide neuroregeneration, consequently enhancing cognition in 5xFAD mice, which may provide a brain‐wide neuroregeneration gene therapy for the treatment of AD.

Acquiring the ability of brain regeneration after injury like a salamander is the ultimate goal in the field of regenerative medicine. Unfortunately, mammals have almost lost this miraculous ability to regenerate neurons after injury.^[^ ^9^, ^10^ ^]^ However, glial cells in the brain still retain the basic “engram” of neuroregeneration. Such as reactive astrocytes surrounding Aβ plaques in the brains of 5xFAD mice exhibit some properties of neural stem cells,^[^ ^35^ ^]^ suggesting that reactive astrocytes may be a desired endogenous source cell for regenerating neurons. Indeed, in vivo, reprogramming of astrocytes into functional neurons, termed astrocyte‐to‐neuron conversion, has been achieved by ectopic expression of neuronal pioneer factors in astrocytes such as NeuroD1.^[^ ^11^, ^15^ ^]^ Recently, NeuroD1‐mediated AtN was challenged by some scientists who claim that astrocyte‐converted neurons do not originate from resident astrocytes but rather from pre‐existing neurons infected with AAV, a phenomenon called “leakage” expression.^[^ ^36^ ^]^ Therefore, to prove the real existence of NeuroD1‐mediated conversion in the 5xFAD mouse brain, we provide several lines of evidence for the real conversion of resident astrocytes. First, we observed many “intermediate” cells that contained both astrocytic and neuronal markers in NeuroD1‐treated 5xFAD mouse brain but not found in the control group. Second, unbiased single‐cell RNA sequencing analysis revealed that typical astrocytic genes were suppressed, whereas neuronal genes were induced during AtN conversion. Notably, this cellular conversion was accompanied by a shift in metabolic preference from glycolysis to oxidative phosphorylation. It is consistent with previous findings that metabolic state is particularly relevant for the AtN conversion, because neurons are highly dependent on oxidative metabolism, while astrocytes rather utilize anaerobic glycolysis.^[^ ^25^, ^26^ ^]^ Third, pseudo‐chronological analysis further revealed that astrocytes with enforced NeuroD1 expression tend to differentiate into neurons. Finally, to exclude the possibility of NeuroD1 broadly changing the specificity of the *Gfap* promoter,^[^ ^36^, ^37^ ^]^ we employed the Cre‐FLEx system, in which the constitutive CAG promoter drives NeuroD1 and reporter gene expression in astrocytes with Cre recombinase. The CAG promoter is a potent constitutive promoter commonly used to drive robust gene expression in mammalian expression vectors. In general, the possibility of NeuroD1 changing promoter specificity through cis‐regulation can be entirely rejected. Together, these results fully demonstrate that NeuroD1 initiates the conversion of astrocytes into neurons, rather than simply leaking expression of the reporter gene in the pre‐existing neurons.

Astrocytes are proliferative and wide‐distribution glial cells in the adult mammalian brain, which share a common developmental origin with neurons.^[^ ^13^ ^]^ Additionally, astrocytes also exhibit limited neurogenic potential in response to invasive injuries such as ischemic stroke, as well as noninvasive conditions like chronic amyloidosis.^[^ ^35^, ^38^ ^]^ Thus, the bias intrinsic characteristics of astrocytes render them optimal internal sources for in vivo neuroregeneration following brain injury or disease. Furthermore, astrocytes have been found to possess similar transcriptional and epigenetic signatures as their neighboring neurons, the fact that direct astrocytes are reprogrammed toward an identity similar to their neighboring neurons.^[^ ^31^ ^]^ In this study, we investigated the NeuroD1‐mediated conversion of astrocytes on the scale of brain‐wide. Specifically, cortical astrocytes were found to be convertible into cortical neurons expressing layer‐specific markers, while hippocampal astrocytes displayed a propensity for conversion into neurons bearing location‐specific markers. These results are in agreement with multiple recent reports showing that astrocyte‐to‐neuron conversion in different brain regions can regenerate distinct neuronal identities despite being induced by the same conversion factors.^[^ ^31^, ^39^, ^40^ ^]^ Most likely, region‐specific astrocytes have inherent properties that influence the outcome of cellular reprogramming, enabling regeneration of diverse neuronal subtypes in a spatially controlled manner, thereby orchestrating the de novo regeneration of neural tissue in situ.

In the scRNA‐seq data, we noticed that the number of GFP^+^ astrocytes in the NeuroD1 group was significantly less than that in the control group. The underlying mechanism for this observation remains unclear, and we propose several potential explanations. First, the process of AtN conversion is accompanied by substantial transcriptomic changes, including the downregulation of genes associated with astrocyte identity.^[^ ^41^, ^42^ ^]^ As an astrocyte‐specific promoter, the GFAP promoter is likely to experience reduced activity during this transition, which may account for the low proportion of GFP^+^ astrocytes observed in the scRNA‐seq data. Furthermore, epigenetic regulation plays a key role in cell reprogramming, with a study suggesting that reprogramming fibroblasts leads to decreased CAG promoter activity early in the cell reprogramming process.^[^ ^43^ ^]^ A similar promoter regulatory mechanism may also exist in NeuroD1‐mediated AtN conversion. Finally, previous research has shown that reprogrammed astrocytes exhibit increased fragility,^[^ ^25^ ^]^ implying GFP^+^ astrocytes reprogrammed by NeuroD1 may more susceptible to loss during tissue digestion. Taken together, these potential mechanisms may contribute to the reduced proportion of GFP^+^ astrocytes in the NeuroD1 group observed in the scRNA‐seq data.

We observed that region‐specific astrocytes exhibit heterogeneity in NeuroD1‐mediated neuronal reprogramming, with high conversion efficiency in the cortex and hippocampus but less efficient in other brain regions such as the olfactory bulbs, thalamus, and brainstem. The discrepancy may be attributed to the varying intrinsic power toward inhibition or promotion of neuronal reprogramming among astrocytes in distinct brain regions. Notch signaling is a well‐known obstacle to neuronal reprogramming in astrocytes,^[^ ^38^, ^44^ ^]^ whose level varies among distinct brain areas, which in turn contributes to disparities in the efficiency of astrocyte conversion induced by the same conversion factor.^[^ ^45^ ^]^ In addition, studies have found that some cortical and hippocampal astrocytes have higher levels of certain transcription factors, such as Sox2 and Ascl1,^[^ ^46^, ^47^ ^]^ which are known to conduct neuronal reprogramming from astrocytes.^[^ ^48^, ^49^ ^]^ Another special result we observed is that NeuroD1‐mediated brain‐wide AtN regeneration did not regenerate GABAergic neurons, but almost exclusively excitatory neurons, which are preferentially vulnerable to damage early in the course of AD.^[^ ^50^ ^]^ NeuroD1 is highly expressed in neural progenitor cells and contributes to the generation of most of the excitatory neurons,^[^ ^51^ ^]^ indicating that NeuroD1 preferentially guides precursor cells to differentiate into glutamatergic neurons. Furthermore, a recent study identified a subpopulation of astrocytes in the hippocampus with a glutamatergic signature. For example, some genes involved in glutamatergic vesicle exocytosis are strongly expressed not only in glutamatergic neurons but also in this subset of astrocytes.^[^ ^52^ ^]^ Overall, these findings suggest that astrocytes prefer to be converted into glutamatergic neurons under NeuroD1‐mediated AtN conversion.

In vivo, glia‐to‐neuron conversion has been shown to significantly relieve symptoms in a variety of CNS disease models, including ischemic stroke, Parkinson's disease, Huntington's disease, epilepsy, spinal cord injury, and retinal lesions.^[^ ^53^ ^]^ However, local regeneration of a limited number of neurons by intracranial injection of conversion vectors may not be applicable in the treatment of AD, as massive neurodegeneration occurs throughout the brain, particularly in the neocortex and hippocampus. Therefore, we developed a brain‐wide AtN conversion gene therapy that can not only regenerate ≈500 000 new neurons but also be widely distributed in the neocortex and hippocampus, which is currently difficult to achieve with other strategies. Moreover, we found strong evidence of functional network integration of converted neurons in the brains of 5xFAD mice. Whole‐cell patch clamp recording revealed that the converted neurons received functional synaptic innervation that contained both excitatory and inhibitory post‐synaptic currents. Retrograde tracing revealed that axons of converted neurons in the frontal cortex project to the ipsilateral BPN nucleus, and axons of converted neurons in the hippocampus project to the contralateral hippocampus. Similar to previous studies, converted neurons can find the correct targets for axonal projections.^[^ ^15^, ^39^, ^54^ ^]^ Most importantly, cognitive behavior tests were significantly improved after treating 5xFAD mice with brain‐wide gene therapy conversion. Next, we used the DREADD‐based chemogenetic method to specifically silence the activity of converted neurons, demonstrating that the activity of converted neurons contributes to cognitive recovery. Together, because astrocytes are widely distributed throughout the brain and possess intrinsic proliferative capacity, our brain‐wide gene therapy conversion may provide an alternative method to regenerate large numbers of new neurons for the treatment of neurodegenerative diseases such as AD.

Moreover, the cognitive improvements in 5xFAD mice following NeuroD1‐mediated AtN gene therapy, despite unchanged excitatory–inhibitory (E–I) balance and Aβ levels, may be attributed to several mechanisms. Notably, the transition from pro‐inflammatory to neuroprotective astrocytes may play an important contribution, as reactive astrocytes have a significant impact on the progression of Alzheimer's disease.^[^ ^55^, ^56^ ^]^ Astrocytes are essential for brain homeostasis, and a reduction in proinflammatory astrocytes, along with an increase in protective ones, may enhance synaptic function, support neuronal survival, and reduce neurotoxic Aβ effects, thus fostering a more favorable environment for cognitive recovery. Additionally, although new excitatory neurons did not alter E–I balance, their integration into existing circuits may enhance synaptic plasticity and network connectivity, which are vital for cognitive functions like learning and memory. These new neurons might compensate for deficits in AD‐affected circuits, improving network dynamics. Furthermore, the AtN conversion could indirectly influence the glial population, triggering protective responses and limiting neurodegeneration. Together, these factors suggest that cognitive recovery results from a combination of enhanced synaptic plasticity, glial modulation, and indirect effects of neurogenesis. Future studies should further explore these mechanisms to better understand the therapeutic potential of NeuroD1 in the mouse model of Alzheimer's disease.

The mouse brain contains ≈75 million neurons, which are interconnected in a complex and ordered manner, establishing functional neuronal circuits that enable the execution of diverse functions. Although we have discovered that the converted neurons in the frontal cortex can form ipsilateral projections, and the neurons in the hippocampus can form contralateral projections, the existence of other projections in the converted neurons remains to be determined. Moreover, the molecular mechanisms guiding the orientation of axonal growth of converted neurons toward downstream target neurons remain unclear, which is the first limitation of this study. Using fMOST whole‐brain imaging combined with stereocytometry, we regenerated ≈500 000 new neurons in the cortex and hippocampus of 5xFAD mice, but this technique was unable to quantify nonfluorescent endogenous neurons. Therefore, it is still unknown whether NeuroD1‐mediated AtN can increase the overall number of neurons in the brains of 5xFAD mice, and it is worth continuing to study in the future. Despite we provide a valuable brain‐wide gene therapy conversion in AD mouse brain by single retro‐orbital injection of AAV‐PhP.eB vectors, this viral vector can only penetrate the BBB of specific mouse strains, which poses a huge obstacle to possible future clinical applications,^[^ ^57^ ^]^ and this is the third limitation. Therefore, new AAV serotypes that can penetrate the BBB in large animals are worthy of further exploration.^[^ ^58^ ^]^ In addition, AAV‐PhP.eB is designed to preferentially infect cells in the CNS,^[^ ^59^, ^60^ ^]^ but systemic injection may result in some degree of off‐target transduction in peripheral tissues, however, we did not examine transduction of AAV‐PhP.eB in peripheral organs in this study. Further investigations into the potential peripheral effects of AAV‐PhP.eB infection, particularly on GFAP^+^ cells outside the brain, would be valuable, and this is the fourth limitation. The fifth obvious limitation of this study is that AtN conversion have no effect on Aβ deposition within the AD mouse brain, which leaves newly generated neurons still exposed to the toxic effects of Aβ aggregates. To achieve better efficacy, combination therapy may be a better choice in the future. For example, combining brain‐wide gene therapy conversion with immunotherapy antibody that targets deposited Aβ,^[^ ^61^ ^]^ can both regenerate neurons while also reducing the deposition of Aβ in the Alzheimer's brain.

In conclusion, the brain‐wide gene therapy approach for AtN conversion offers a promising avenue for addressing the complex challenges posed by AD. By focusing on regenerating a large number of neurons throughout the brain, rather than just local regions, we expect to revitalize neural circuits and restore cognitive functionality in patients with AD. Future research should continue refining this method, optimizing its delivery, and increasing its efficiency to maximize therapeutic benefits for those affected by this debilitating disorder.

## 4 Experimental Section

### Mice

The transgenic mouse strains used in this study include GFAP::Cre [B6.Cg‐Tg(Gfap‐cre)77.6Mvs/2J, stock number: 024098] and 5xFAD transgenic mice(stock number: 006554), carrying mutations on both human APP (Swedish (K670N/M671L), Florida (I716V) and London (V717I) and human PS1 proteins (M146L and L286V) all purchased from The Jackson Laboratory. The GFAP::Cre mice were crossed with the 5xFAD transgenic mice for breeding. The offspring at the age of 6–9 months (5xFAD/GFAP::Cre and age‐matched WT littermates) were used in the present study. All mice were subjected to a standard 12 h light and dark cycle with sufficient water and food. Experimental protocols were approved by the Laboratory Animal Ethics Committee of Jinan University, China (approval No. IACUC‐20200916‐03) and the Pennsylvania State University IACUC, USA (approval No. 46803).

### AAV Production

AAV used for AtN conversion include AAV9 GFAP::NeuroD1‐P2A‐GFP/GFAP::GFP and AAV PhP.eB CAG::FLEx‐NeuroD1‐P2A‐GFP/CAG::FLEx‐GFP were produced by PackGene Biotech, LLC (Guangzhou, China). GFAP1.6 (length 1677 bp) promoter was used in this study. The production steps of AAV mentioned above entail the following: Initially, a considerable amount of highly purified plasmid was extracted and co‐transfected into HEK293 cells. Subsequently, AAV was concentrated and purified through Iodixanol gradient (Merck/Sigma‐Aldrich, St Louis, MO, USA) ultracentrifugation. Then the virus titer was measured by FWD ITR and REV ITR primers for specific detection of the AAV vector's ITR sequence using SYBR green qPCR. The purity of AAV was assessed through SDS‐PAGE electrophoresis. An endotoxin test was conducted via gel assay, yielding a result of less than 10 EU mL^−1^. The purity of capsid protein was determined by SDS‐PAGE, revealing the absence of any noticeable impurities. For chemogenetic inhibition, AAV PhP.eB DIO‐hSyn::hM4Di‐mCherry (Addgene #44362, 2.3 × 10^13^ GC mL^−1^) was used in this study. AAV titer adjustment was performed using PBS containing 0.001% F‐68 (Poloxamer 188 Solution, Caisson Laboratories, Smithfield, UT, USA). All AAVs were stored in a −80 °C freezer and used within 12 months of production.

### AAV and CTB Injection

Brain surgeries were conducted on 6–6.5 months old mice. For AAV9 injection, the mice were anesthetized by intraperitoneal injection of 20 mL kg^−1^, 1.25% Avertin (Sigma, T48402). Shaving the hair and washing the scalp with iodine tincture and ethanol, then the mice were placed into a stereotaxic setup. The operation began with a midline scalp incision followed by the creation of a (≈1 mm) drill hole on the skull and then AAV9 were injected into both sides of the mouse cortex (AP + 0.6 mm, ML ± 2 mm, DV − 0.7 mm) and hippocampus (AP − 2 mm, ML ± 1.5 mm, DV − 1.3 mm) (1 µL, 1 × 10^12^ GC mL^−1^). After the viral injection, the needle was kept in place for at least 10 min before being slowly withdrawn. The stereotaxic coordinates were measured from bregma.

The mice were anesthetized by Isoflurane (3%) and then AAV PhP.eB CAG::FLEx‐ NeuroD1‐GFP/ CAG::FLEx‐GFP were delivered into the vein through retro‐orbital injections (5 µL, 1 × 10^13^ GC mL^−1^). Mice were put back in their original cage after awakening. For retrograde labeling of the converted neurons, CTB‐555 (500 nL) was injected into BPN (AP − 4 mm, ML + 0.4 mm, DV − 5.5 mm) and hippocampus (AP − 2 mm, ML −1.5 mm, DV − 1.3 mm) two months after injection of the AAV PhP.eB‐NeuroD1 virus. After the CTB injection, the pipette was held for 10 min before withdrawal. The slice staining was performed 1–2 weeks after CTB injection.

### Immunohistochemistry and Analysis

Mice were deeply anesthetized with 1.25% Avertin and then sequentially perfused with ice‐cold artificial cerebrospinal fluid (aCSF) to wash away the blood. Then 4% paraformaldehyde (PFA) was used to fix the brain overnight. After fixation, the brain was cut into 30 µm sections by a vibratome (Leica, VTS1200). For immunostaining, brain slices were washed with PBS and blocked for 2 h at room temperature in the blocking solution (10% normal donkey serum, 5% bovine serum albumin, and 0.2% Triton X‐100 prepared in PBS). Primary antibodies were diluted in the blocking buffer and incubated at 4 °C for 24–48 h. After 3 washes with 0.2% PBST (0.2% tween‐20 in PBS), the brain slices were incubated with secondary antibodies conjugated to Alexa Fluor 488, Alexa Fluor 555, or Alexa Fluor 647 (1:1000) and 0.5 mg ml^−1^ DAPI (F. Hoffmann‐La Roche, Natley, NJ, USA) in blocking buffer for 2 h at room temperature and washed with PBS (12 min × 3 times). Finally, samples were mounted with VECTASHIELD mounting medium (VECTOR Laboratories, Burlingame, CA, USA) and sealed with nail polish. Representative images were taken with a Zeiss Axioplan fluorescent microscope (Axio Imager Z2, Zeiss, Göttingen, Germany) or confocal microscope (LSM880, Zeiss, Jena, Germany).

Primary antibodies used were listed as follows: mouse anti‐GFAP (1:1000, Cat# G3893, Sigma), rabbit anti‐GFAP (1:500, Cat# G9269, Sigma), rabbit anti‐NeuN (1:1000, Cat# ab177487, Abcam), guinea pig anti‐NeuN (1:2000, Cat# ABN90, Millipore), rabbit anti‐S100β (1:500, Cat# ab52642, Abcam), rabbit anti‐NeuroD1 (1:1000, Cat# ab205300, Abcam), rat anti‐Ctip2 (1:200, Cat# ab18465, Abcam), mouse anti‐CUX1 (1:500, Cat# ab54583, Abcam), Chicken anti‐GFP (1:1000, Cat# ab13970, Abcam), mouse anti‐Aβ42 (1:1000, Cat# 800701, biolegend), rabbit anti‐Iba1 (1:1000, Cat# #019‐19741, Wako), rabbit anti‐Olig2 (1:1000, Cat# AB9610, Millipore), rabbit anti‐NG2 (1:500, Cat# AB5320, Millipore), rabbit anti‐Prox1 (1:500, Cat# AB5475, MillipoDoure), mouse anti‐GAD67 (1:1000, Cat# MAB5406, Millipore).

### Electrophysiology

Brain samples were prepared two months after the virus injection. Mice were anesthetized with 1.25% Avertin and perfused with ice‐cold cutting solution (in mM: 93 NMDG, 2.5 KCl, 30 NaHCO_3_, 1.25 NaH_2_PO_4_, 15 glucose, 20 HEPES, 12 N‐Acetyl*‐L‐*cysteine, 5 sodium ascorbate, 2 thiourea, 3 sodium pyruvate, 7 MgSO_4_, 0.5 CaCl_2_, pH 7.35–7.45, 300–310 mOsm, solution was bubbled with 95% O_2_/5% CO_2_). After decapitation, the brains were rapidly transferred into an ice‐cold oxygenated cutting solution and cut into 300 µm thick coronal sections with a vibratome (Leica, VTS1200). Then, slices were transferred to holding solution with continuous 95% O_2_/5% CO_2_ bubbling (in mM: 92 NaCl, 2.5 KCl, 1.25 NaH_2_PO_4_, 30 NaHCO_3_, 15 glucose, 20 HEPES, 12 N‐Acetyl‐*L*‐cysteine, 5 sodium ascorbate, 2 thiourea, 3 sodium pyruvate, 2 MgSO_4_, 2 CaCl_2_). After 1 h recovery, the slices were transferred to a recording chamber that was filled with artificial cerebral spinal fluid (in mM: 124 NaCl, 2.5 KCl, 26 NaHCO_3_, 1.25 NaH_2_PO_4_, 2.5 CaCl_2_, 1.3 Mg SO_4_ and 10 glucose, and constantly bubbled with 95% O_2_/5% CO_2_). Whole‐cell recordings were performed using a pipette solution consisting of 125 mm K‐Gluconate, 5 mm Na‐phosphocreatine, 10 mm KCl, 2 mM EGTA, 10 mM HEPES, 4 mm MgATP, and 0.5 mm Na_2_GTP (pH 7.3, adjusted with KOH, 280–290 mOsm L^−1^). To record the spontaneous synaptic events, the potassium gluconate in the pipette solution was replaced with Cs‐methanesulfonate to block K^+^ channels and reduce noise. Pipette resistance was typically 4–6 MΩ. The membrane potential was held at −70 mV for sEPSC recording, and at 0 mV for sIPSC recording. Data were collected using pClamp 10 and Clampex 10.4 software (Molecular Devices, Palo Alto, CA), and sampled at 10 kHz and filtered at 3 kHz, analyzed with Clampfit 10.4.

### fMOST Whole Brain Imaging and Quantification

Mice were anesthetized and perfused with ice‐cold saline solution and 4% paraformaldehyde (PFA), sequentially. Brains were post‐fixed in 4% PFA for 24 h, and transferred into 0.01 m PBS, and rinsed at 4 °C overnight. Then the fixed brains got dehydrated in graded ethanol solutions. Afterward, each brain was impregnated with graded LR White (Ted Pella Inc, 18182 1KT) under dark conditions in 4 °C. Finally, individual brains were placed into capsules filled with LR White, and these capsules were set in a vacuum oven for 24 h in 37.5 °C. After embedding, the brain‐wide fluorescence micro‐optical sectioning tomography (fMOST) via structured illumination is employed (Biomapping5000, OEBio Inc, Wuhan, China). The brain samples were then sectioned coronally at an interval of 2 µm in an anteroposterior (AP) direction to achieve the axial scan, counterstained in PI, and then underwent fluorescence image acquisition via mosaic scan. The images had a voxel size of 0.32 × 0.32 × 2 µm, enabling 3D reconstruction. The raw data acquired by the BPS necessitated mosaic stitching and illumination correction image pre‐processing. Then, the pre‐processed data were visualized and converted to figures using Imaris software (v.9.8.0, Oxford Instrument plc, Abingdon, UK).

To quantify the total number of converted neurons (GFP^+^) in the entire cortex and hippocampus of mice, Imaris software was used. First, the software with some confocal images of regenerating neurons stained with NeuN was provided and continuously adjusted the software's identification parameters until more than 90% of GFP^+^ cells in the confocal images were co‐labeled with NeuN. Then, these parameters were loaded into the software to label the GFP cells in the confocal images, and it was verified that more than 90% of GFP^+^ cells were NeuN‐positive. Finally, the parameters of cortical neurons were set to 15 µm, and hippocampal neuron parameters were set to 10 µm. The authors used the Surfaces‐Mask function in Imaris to separate the hippocampus and cortex respectively, and then applied the spots function to count the number of neurons in the hippocampus and cortex respectively. Snapshot and Animation functions in Imaris were applied to make photos.

### Single‐Cell RNA Sequencing Library Preparation

To obtain fresh brain tissue, the mice were anesthetized with 1.25% Avertin and then sequentially perfused with ice‐cold aCSF to wash away the blood. The fresh hippocampus was removed with tweezers and then dissociated into single‐cell suspension using the papain kit (Worthington) according to the manufacturer's protocol. The cell suspension was injected into the SCOPE‐chip microfluidic chip (Singleron) to complete the separation of single cells based on the principle of “Poisson distribution”. Cells fell into specially customized chip microwells under the influence of gravity, ensuring that only one cell fell into each microwell. Millions of magnetic beads carrying unique cell tags (Cell Barcode) were then added to the microwells of the chip to ensure that only one magnetic bead fell into each microwell. After the cells were lysed, magnetic beads with unique cell tags (Barcode) and molecular tags (UMI) captured the mRNA by binding to the poly(A) tail on the mRNA, and label the cells and mRNA. The magnetic beads in the chip were collected, and the mRNA captured by the magnetic beads was reverse transcribed into cDNA and amplified. After cDNA fragmentation, adapter ligation, and other necessary steps, a sequencing library suitable for the Illumina sequencing platform was constructed.

### Primary Analysis of Raw Read Data

Raw reads were processed with fastQC and fastp to remove low‐quality reads. Poly‐A tails and adaptor sequences were removed by Cutadapt. After quality control, reads were mapped to the reference genome using STAR. Gene counts and UMI counts were acquired by FeatureCounts software. Expression matrix files for subsequent analyses were generated based on gene counts and UMI counts.

### Quality Control, Dimension‐Reduction and Clustering (Scanpy)

Scanpy v1.8.2 was used for quality control, dimensionality reduction, and clustering under Python 3.7. For each sample dataset, the expression matrix was filtered by the following criteria: 1) cells with a gene count less than 200 or with a top 2% gene count were excluded; 2) cells with a top 2% UMI count were excluded; 3) cells with mitochondrial content >20% were excluded; 4) genes expressed in less than 5 cells were excluded. After filtering, cells were retained for the downstream analyses. The raw count matrix was normalized by total counts per cell and logarithmically transformed into a normalized data matrix. The top 2000 variable genes were selected by setting flavor = ‘seurat’. Principle Component Analysis (PCA) was performed on the scaled variable gene matrix, and the top 20 principles components were used for clustering and dimensional reduction. Cells were separated into 13 clusters by using the Louvain algorithm. Cell clusters were visualized by using Uniform Manifold Approximation and Projection (UMAP). The cell type identity of each cluster was determined with the expression of canonical markers found in the DEGs using the SynEcoSys database. Heatmaps/dot plots/violin plots displaying the expression of markers used to identify each cell type were generated by Seurat v3.1.2 DoHeatmap/DotPlot/Vlnplot.

### Differentially Expressed Genes (DEGs) Analysis (scanpy)

To identify differentially expressed genes (DEGs), the scanpy.tl.rank_genes_groups function based on the Wilcoxon rank sum test with default parameters was used, and selected the genes expressed in more than 10% of the cells in either of the compared groups of cells and with an average log (Fold Change) value greater than 0.25 as DEGs. Adjusted *p‐*value was calculated by benjamini‐hochberg correction and the value 0.05 was used as the criterion to evaluate the statistical significance.

### Pathway Enrichment Analysis

To investigate the potential functions of the changed gene, the Gene Ontology (GO) was used with the “clusterProfiler” R package 3.16.1. Pathways with *a p*‐value less than 0.05 were considered as significantly enriched. Gene Ontology gene sets including molecular function (MF), biological process (BP), and cellular component (CC) categories were used as reference.

### Single‐Cell Trajectory and Entropy Analysis

To map differentiation/conversion of astrocyte to neuron, pseudotime trajectory analysis was performed with Monocle2. For constructing the trajectory, highly variable genes were selected from the cluster by Seurat v3.1.2 FindVairableFeatures, and dimension‐reduction was performed by DDRTree. The trajectory was visualized by plot_cell_trajectory.

### Cell Pluripotency Analysis

To study cell pluripotency, as shown in previously published studies, four algorithms including StemID,^[^ ^19^ ^]^ CytoTrace,^[^ ^21^ ^]^ SLICE,^[^ ^20^ ^]^ and CCAT^[^ ^18^ ^]^ were employed. StemID is an algorithm based on the projection of cells and Shannon entropy for the inference of differentiation trajectories and the prediction of the stem cell identity. StemID assign every cell onto a specific link between its cluster of origin and another cluster based on the longest projection of the vector connecting the cluster center with the cell position onto these links. The significance of each link was calculated by repeating the process of randomly assigning cell positions 1000 times in the embedded space. The ensemble of significant links was used to construct lineage trees as potential differentiation trajectories. Meanwhile, the specific differentiation direction was determined by calculating the transcriptome entropy of cells. CytoTRACE v0.3.3 (a computational method that predicts the differentiation state of cells from single‐cell RNA‐sequencing data using gene counts and expression) was used to predict the differentiation potential of cell subpopulations. SLICE (version 0.99.0) was used to evaluate the stemness of cells by the entropy of gene expression based on single‐cell expression profiles. After removing ribosomal genes, an SLICE object was created to perform the bootstrap calculation of single‐cell gene entropy values by the getEntropy function. Correlation of Connectome and Transcriptome (CCAT) could return accurate single‐cell potency estimates of a million cells. CCAT was an ultrafast method for estimating the differentiation capacity of single cells from scRNA‐Seq data that was scalable to upcoming scRNA‐Seq studies analyzing millions of cells. CCAT measurements were directly driven by the previously proposed entropy rate (SR). Since the dynamic range of the local entropy Si was smaller, this meant that the SR could be further approximated in scale by the dot product of transcriptome x and connectome k. Therefore, CCAT was defined by the Pearson correlation coefficient (PCC) of connectome k and transcriptome x.

### RNA Velocity Analysis

For RNA velocity analysis, a loom file containing 5719 cells and the GRCm38 (mm10) reference genome were utilized. The analysis was conducted using the velocyto (ref) and scVelo (ref) tools in Python, with default parameters applied.^[^ ^28^ ^]^ To ensure visualization consistency, the results were projected onto a UMAP plot derived from Seurat clustering analysis.

### Chemogenetical Manipulation

To manipulate newly converted neural activity, the virus AAV PhP.eB CAG::FLEx‐NeuroD1‐P2A‐GFP /CAG::FLEx‐GFP (5 µL, 1 × 10^13^ GC mL^−1^) and AAV PhP.eB DIO‐hSyn::hM4Di‐mCherry (5 µL, 1 × 10^13^ GC mL^−1^) were mixed to 10 µL in a 1:1 ratio. Then the mixture was retro‐orbitally injected into 5xFAD/GFAP‐Cre mice. Following tests were administered 2 months after virus injection to elucidate the influence of AtN conversion. For electrophysiology, whole cell patch clamp recordings were performed, and CNO (Cat#34233‐69‐7, Sigma‐Aldrich) was reconstituted in 5% DMSO and was used with a concentration of 5 µm in aCSF, which was perfused at 2–3 mL min^−1^ after a baseline recording was obtained. For the contexture fear conditioning test, mice were intraperitoneally injected CNO (5 mg kg^−1^) 15 min before the electric shock, and the same volume of saline was given as the vehicle control.

### Behavioral Tests and Analysis

Mice were moved to the behavioral testing room one hour in advance to acclimate before starting the behavioral test. All behavioral tests were conducted during the light phase of the light/dark cycle, and all behaviors were performed and analyzed by two investigators who were blinded to the treatment conditions of the animals.

### Y Maze Test

Spontaneous alternation performance was conducted following the protocol described in the previous study.^[^ ^17^ ^]^ Each mouse was placed in the center of a symmetrical Y‐maze and allowed to freely explore the maze for an 8‐minute session. The first 2 min were excluded from data analysis because the mice were still exploring the new environment. The total number and sequence of arm entries made during the last 6 min were recorded and analyzed. An arm entry was defined as when the hind paws of the mice had been completely placed in the arm. The spontaneous turn behavior score = total number of turns/maximum number of turns × 100%.

### Odor Habituation Test

The odor habituation test was conducted to evaluate the ability of mice to remember odors, as previously described.^[^ ^16^ ^]^ Four different odors (heptanone, isoamyl acetate, limonene, and ethyl valerate; Sigma) were diluted in mineral oil at a ratio of 1:1000 and applied to a cotton‐applicator stick, which was then enclosed in a plastic tube. The odor stick was inserted into a port on the side of the animal's home cage, and the odors were presented for four successive trials (one block), each lasting 20 s, with a 30‐second inter‐trial interval. The duration of time spent investigating was defined as snout‐oriented sniffing within a proximity of 1 cm from the odor presentation port, and it was recorded across all trials by a single observer who was blinded to the mouse groups. Mice were tested in a counter‐balanced order. A mouse with intact memory was expected to spend decreasing time investigating (sniffing) the odor stick, which would indicate learning and memory ability. It is expected that mice with intact learning ability would exhibit a progressive decrease in investigation time, specifically in terms of sniffing behavior directed toward the odor stick. Such behavior would serve as an indicator of successful learning and memory retention.

### Contextual Fear Conditioning Test

Mice were exposed to a novel context, consisting of an observation chamber with defined lightning, odor, background noise, and geometry. The chamber was a box made of PVC plastic that contained a removable foot shock grid and sensors for the animal's movements. Each mouse was allowed to habituate the new chamber for 5 min on day 1. On day 2, the mouse was placed into the chamber for 3 min to record the baseline movement of the mice, then mice were received 3 repetitions of a mild electric foot shock (0.6 mA, lowest power shock) for 2 s, at 58 s inter‐trial intervals. On day 3, each mouse was placed into the same chamber for 3 min and the freezing time was recorded to evaluate the memory ability.

### Morris Water Maze Test

The Morris water maze apparatus consisted of a large circular pool (120 cm in diameter) filled with opaque 21 ± 1 °C water with nontoxic white pigment. Visual cues were adhered to the inner walls of the pool. The whole procedure consisted of 5‐day acquisition training and a probe trial on the next day. Initially, acquisition training was performed, with a platform of fixed position submerged 1.5 cm below the surface. The mouse was gently put into the water with its head facing the wall of the pool, and the drop position was semi‐random between trials. Mice received two training sessions with a 3‐hours intersession interval for 5 continuous days. Every session consisted of two trials with 10 min intertrial intervals. During each trial, the time spent by an individual mouse to find the platform was recorded, and it was allowed to stay on the platform for 10 s once the mouse found it. The maximum time of each trial was 60 s. If a mouse failed to find the platform, it was guided to the platform and then allowed to sit on it for another 10 s. For the probe trial, the platform was removed and each mouse were allowed to swim for 60 s, with a drop location 180° from the original platform. After each trial, mice were wiped off the excess water and dried off with an electric hot‐air blower before being placed back into their home cage.

### Statistical Analysis

Data were shown as mean ± standard deviation (SD) or box plot. Statistical significance was tested with Student's two‐tailed paired or unpaired *t*‐tests and One Way ANOVA test followed with Tukey post hoc test or Fisher's LSD test. The significance level was set to *P *< 0.05. Statistical analysis was performed with GraphPad Prism 7.0.

## Conflict of Interest

Gong Chen is a co‐founder of NeuExcell Therapeutics Inc.

## Author Contributions

Z.W. and L.X. contributed equally to this work. Z.W. and G.C. supervised and designed the research. Z.W., L.X., Y.X., A.S., S.S., W.‐Y.J., Z.‐R.L., and Y.‐W.G. completed most of the experiments. Z.W., L.X., and G.C. analyzed the data and made the figures. L.X. performed bioinformatics analysis. X.L. and Z.‐F.L. performed fMOST study. Z.W., G.C., L.X., and Y.X. wrote the manuscript with input from all authors.

## Supporting information



Supporting information

## Data Availability

The data that support the findings of this study are available on request from the corresponding author. The data are not publicly available due to privacy or ethical restrictions.
