# Supplementary material for: Brain‐Wide Neuroregenerative Gene Therapy Improves Cognition in a Mouse Model of Alzheimer's Disease
Source: Adv Sci (Weinh). 2025 Feb 14;12(14):2410080. doi: 10.1002/advs.202410080 (PMC11984881; doi:10.1002/advs.202410080)
Supplement: Supplementary file 1 — Supporting information [file ADVS-12-2410080-s001.pdf]

ADVANCED SCIENCE Open Access

Supporting Information

for *Adv. Sci.*, DOI 10.1002/advs.202410080

Brain-Wide Neuroregenerative Gene Therapy Improves Cognition in a Mouse Model of Alzheimer's Disease

Zheng Wu, Liang Xu, Yu Xie, Abhijeet Sambangi, Shreya Swaminathan, Zifei Pei, Wenyu Ji, Zeru Li, Yaowei Guo, Zhifei Li and Gong Chen**

Supplementary information

Brain-Wide Neuroregenerative Gene Therapy Improves Cognition in a Mouse Model of Alzheimer's disease

Zheng Wu[*], *Liang Xu, Yu Xie, Abhijeet Sambangi, Shreya Swaminathan, Zifei Pei, Wenyu Ji, Zeru Li, Yaowei Guo, Zhifei Li, Gong Chen*[*]

Z. Wu, L. Xu, Y. Xie, W.Y. Ji, Z.R. Li, Y.W. Guo, Z.F. Li G. Chen

State Key Laboratory of Bioactive Molecules and Druggability Assessment, Guangdong Basic Research Center of Excellence for Natural Bioactive Molecules and Discovery of Innovative Drugs, Key Laboratory of CNS Regeneration (Ministry of Education), Guangdong Key Laboratory of Non-Human Primate Research, GHM Institute of CNS Regeneration, Jinan University, Guangzhou 510632, China

Email: zhengwu@jnu.edu.cn; gongchen@jnu.edu.cn

Z. Wu, A. Sambangi, S. Swaninathan, Z.F. Pei, G.Chen

Department of Biology, Huck Institutes of Life Sciences, Pennsylvania State University, University Park, PA 16802, USA

Supplemental Figures and Legends:

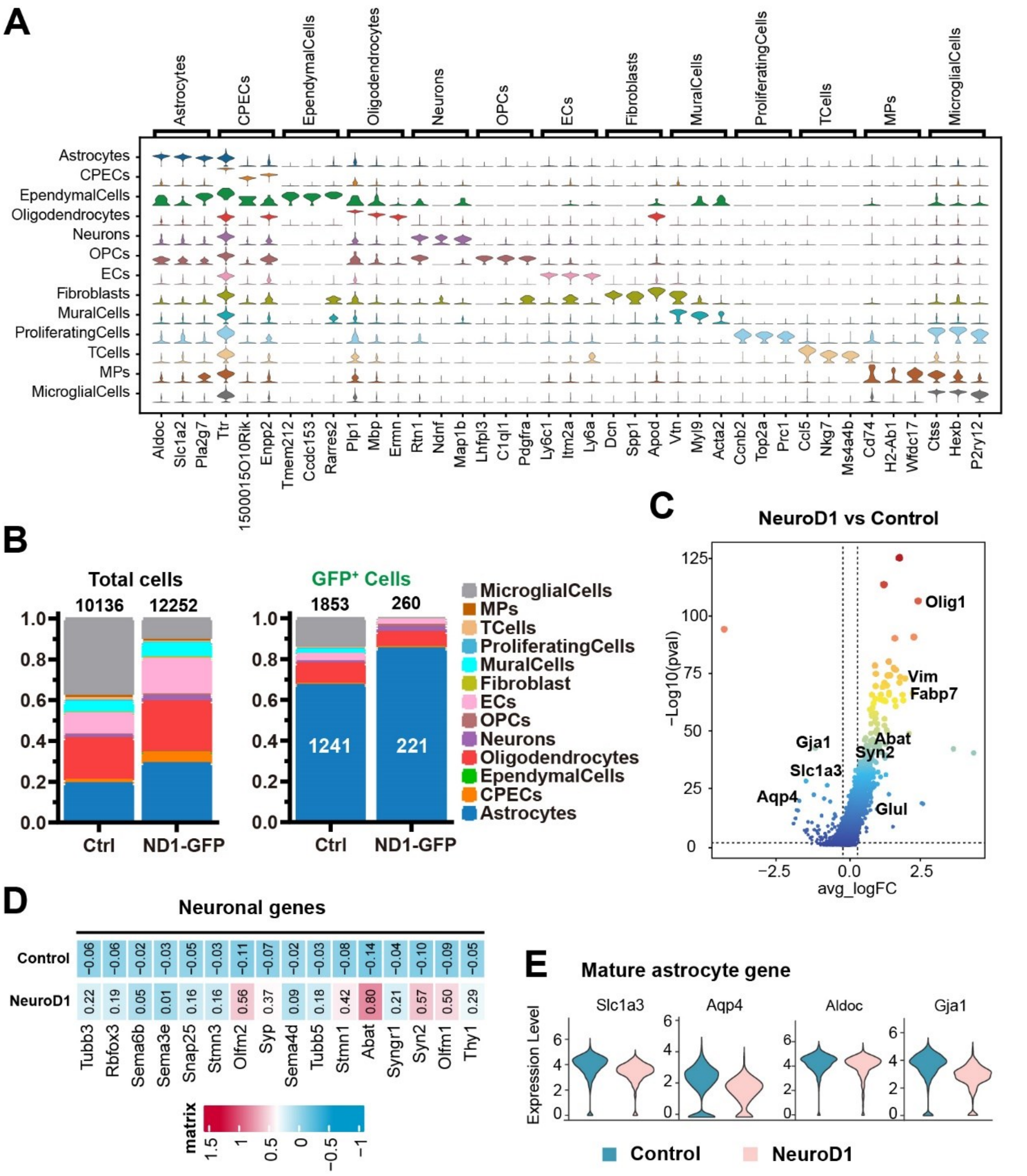

Figure S1. scRNA-seq uncovered the transcriptional program during NeuroD1-induced AtN conversion. A, A panel of marker genes were used to identify Astrocytes, Choroid plexus epithelial cells (CPECs), Ependymal cells (EpendymalCells), Oligodendrocytes, Neurons, Oligodendrocyte precursor cells (OPCs), Endothelial cells (ECs), Fibroblasts, Mural cells, Proliferating cells (ProliferatingCells), T cells, Mononuclear phagocytes (MPs), Microglial cells. **B,** the ratio of different cell types in total cells (left) and GFP$^+$ cells (right). The white numbers in the right bar graph indicate that 1241 and 221 GFP$^+$ astrocytes were found in the control and NeuroD1 groups, respectively. **C,** Volcano plot of up and downregulated genes in the NeuroD1-GFP$^+$ astrocyte group compared to control GFP+ astrocyte group. **D,** The up-regulated

neuronal genes are shown by heatmap. **E,** Violin plot showed that NeuroD1 downregulated mature astrocyte genes.

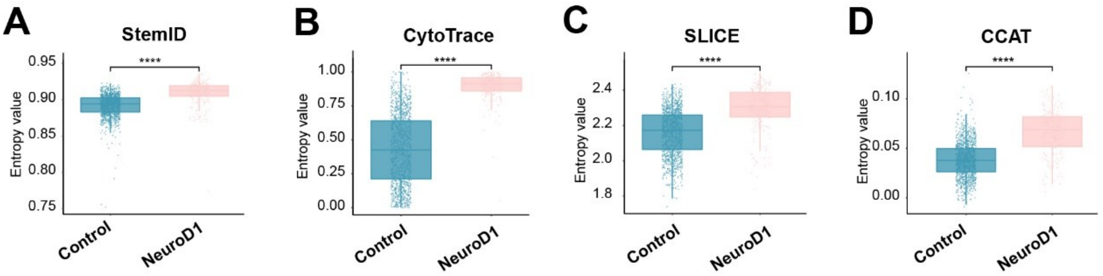

Figure S2. NeuroD1 enhanced astrocyte stemness. A-D, Stemness gene expression analysis using four computational techniques StemID (**A**), CytoTrace (**B**), SLICE (**C**) and CCAT (**D**), showed a significant enhancement of the differentiation capacity.

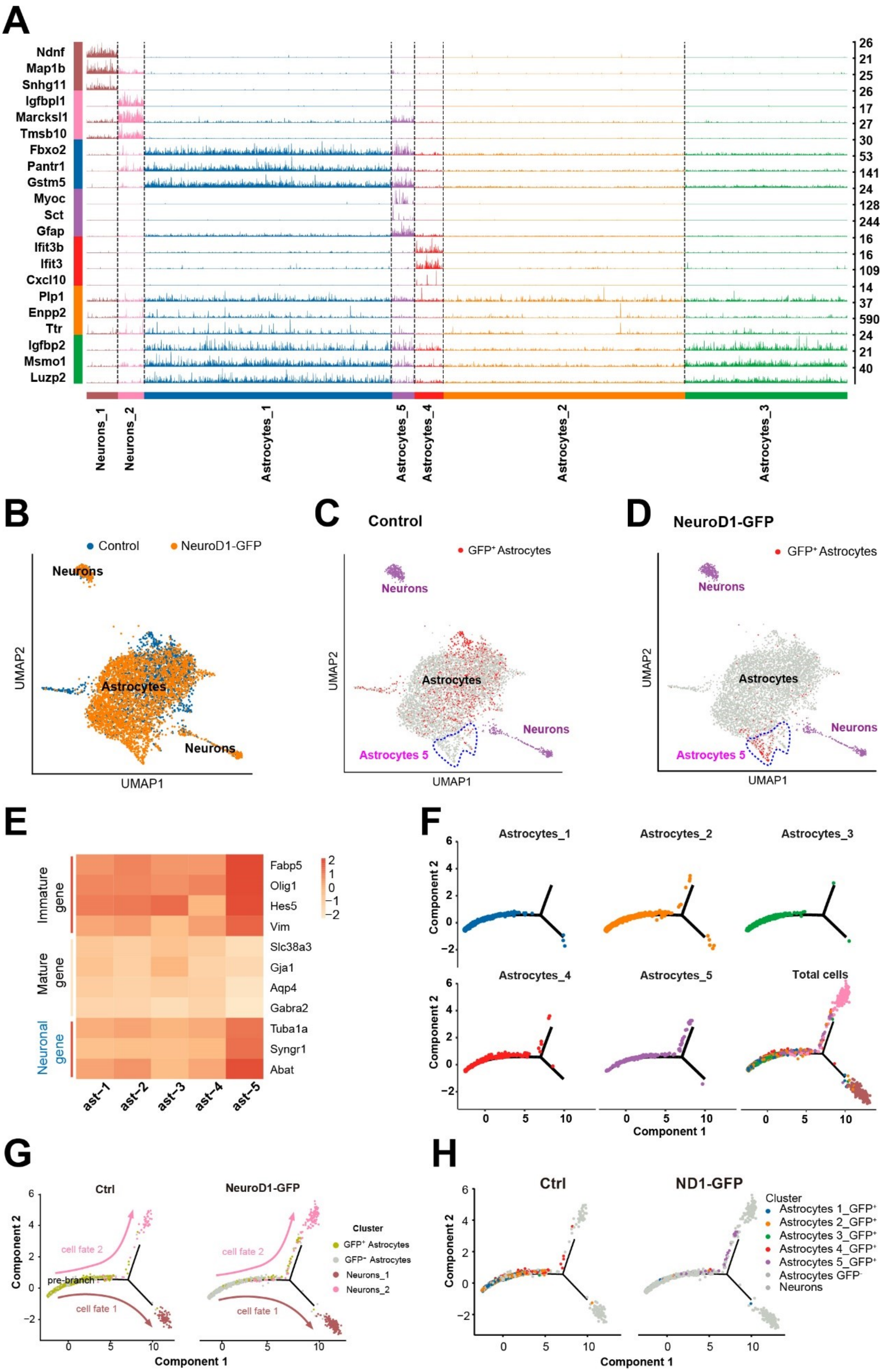
A
Ndnf
Map1b
Snhg11
Igfbpl1
Marcksl1
Tmsb10
Fbxo2
Pantr1
Gstm5
Myoc
Sct
Gfap
Ifit3b
Ifit3
Cxcl10
Plp1
Enpp2
Ttr
Igfbp2
Msmo1
Luzp2
26
21
25
26
17
27
30
53
141
24
128
244
16
16
109
14
37
590
24
21
40
Neurons_1
Neurons_2
Astrocytes_1
Astrocytes_5
Astrocytes_4
Astrocytes_2
Astrocytes_3
B
Control
NeuroD1-GFP
Neurons
Astrocytes
Neurons
UMAP2
UMAP1
C
Control
GFP⁺ Astrocytes
Neurons
Astrocytes
Neurons
Astrocytes 5
UMAP2
UMAP1
D
NeuroD1-GFP
GFP⁺ Astrocytes
Neurons
Astrocytes
Neurons
Astrocytes 5
UMAP2
UMAP1
E
Immature gene
Mature gene
Neuronal gene
Fabp5
Olig1
Hes5
Vim
Slc38a3
Gja1
Aqp4
Gabra2
Tuba1a
Syngr1
Abat
2
1
0
−1
−2
ast−1
ast−2
ast−3
ast−4
ast−5
F
Astrocytes_1
Astrocytes_2
Astrocytes_3
Astrocytes_4
Astrocytes_5
Total cells
Component 2
Component 1
G
Ctrl
NeuroD1-GFP
cell fate 2
pre-branch
cell fate 1
Cluster
GFP⁺ Astrocytes
GFP⁻ Astrocytes
Neurons_1
Neurons_2
Component 2
Component 1
H
Ctrl
ND1-GFP
Cluster
Astrocytes 1_GFP⁺
Astrocytes 2_GFP⁺
Astrocytes 3_GFP⁺
Astrocytes 4_GFP⁺
Astrocytes 5_GFP⁺
Astrocytes GFP⁻
Neurons
Component 2
Component 1

Figure S3. scRNA-seq showed that NeuroD1-expressed astrocytes were transdifferentiating toward neurons. **A,** A panel of top 3 genes showing the sub-cluster of Neurons and Astrocytes. **B,** Distribution of astrocytes and neurons in UMAP of control (blue dots) and NeuroD1 (orange dots) treated mice. **C-D,** Scatter plots of GFP^+ astrocytes (red dots) in UMAP from control (**C**) and NeuroD1-treated (**D**) mice. The dashed boxes indicate subpopulations of astrocytes 5. **E,** Representative the relative expression of immature astrocyte gene, mature astrocyte gene and neuronal gene in astrocyte cluster 1 to cluster 5. **F,** Pseudotime analysis of the different clusters of astrocyte and neuron. **G,** Pseudo-time trajectory analysis showed that NeuroD1-GFP^+ astrocytes were more prone to differentiate into the cluster 2 neuronal population (cell fate 2 direction), while GFP^- or control group astrocytes (both GFP^+ and GFP^-) were distributed along within pre-branches. Few astrocytes were distributed along with the cell fate 1 direction in both groups. **H,** Pseudotime analysis showed the clusters of GFP^+ astrocytes, GFP^- astrocytes and neuron.

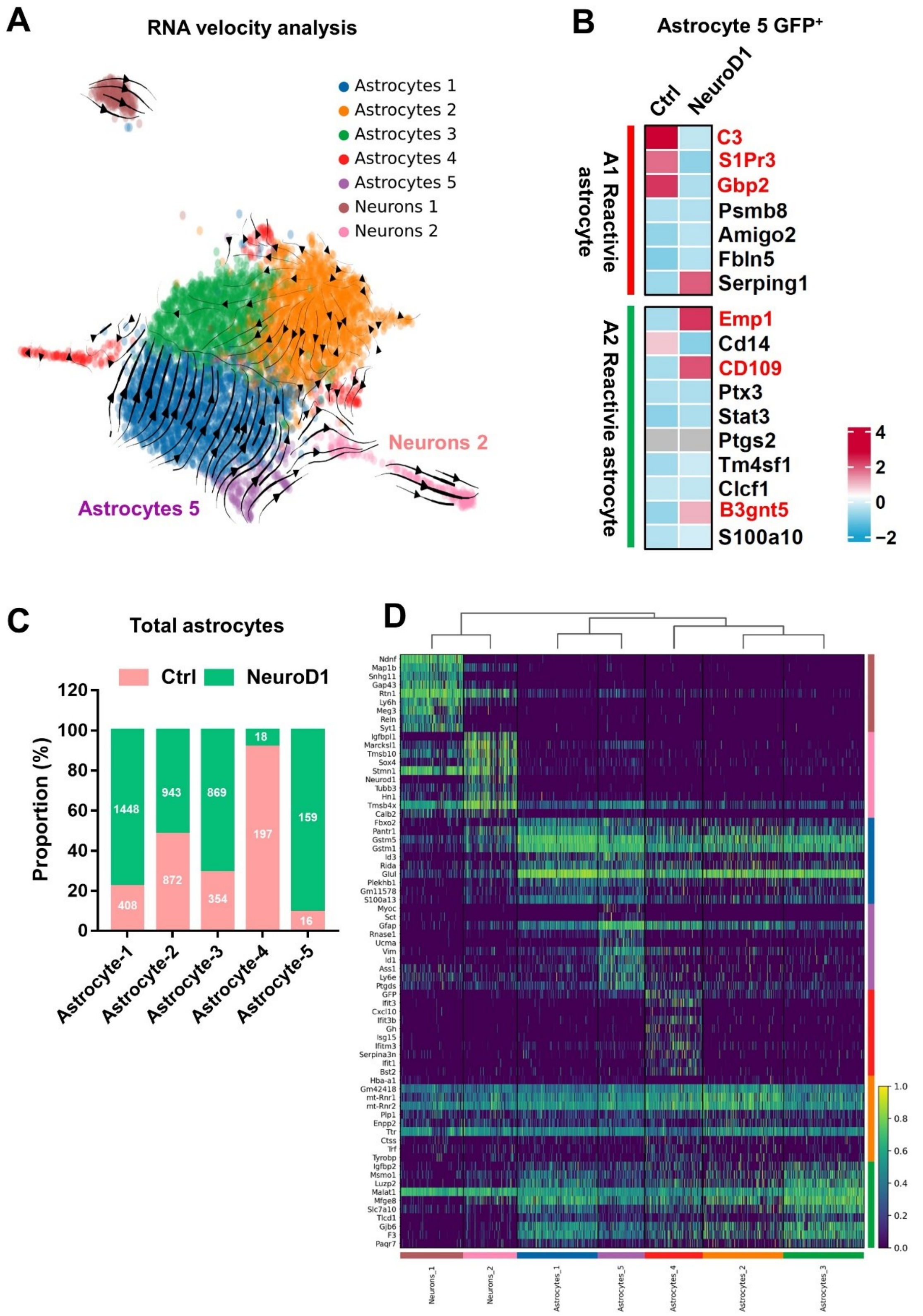

Figure S4. Analysis of cell subcluster proportions in control and NeuroD1-treated mice. A, Velocity streams calculated with CellRank on our subsampled astrocytes and neurons. **B,** Heatmap of typical DEGs in A1 and A2 reactive astrocytes. **C,** Bar graph showing the percentage of different subclusters of astrocytes. The numbers in the bars

indicate the real number of astrocytes in control (bottom) and NeuroD1 (top) groups, respectively. **D,** Heatmap showing the top 10 marker genes for each of the 7 clusters.

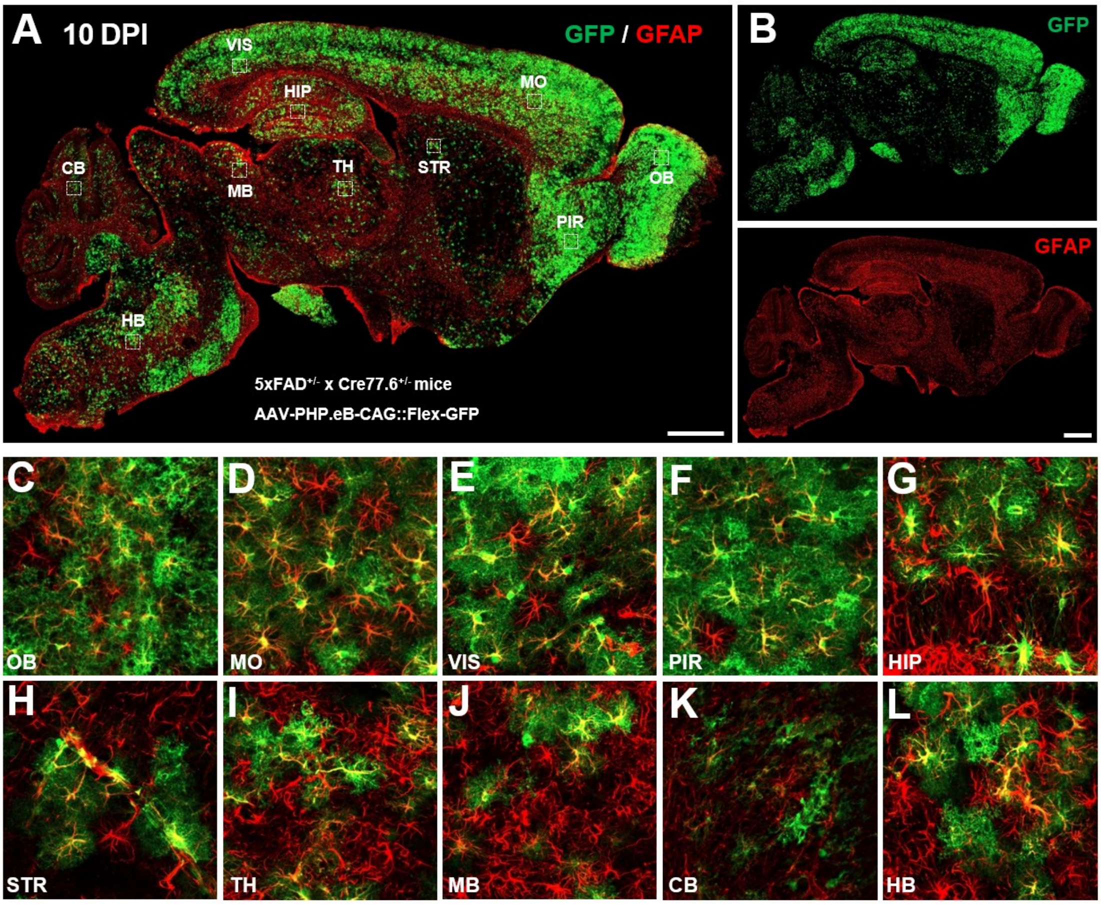

Figure S5. Brain-wide targeting astrocyte in 5xFAD mouse brain by systemic injections of AAV-PHP.eb. A, Typical sagittal view of 5xFAD$^{+/-}$ x Cre77.6$^{+/-}$ bigenic mouse brain 10 days post injection of AAV-PHP.eb-CAG::Flex-GFP (R.O.). GFP was widely expressed in the whole brain. The white dashed box indicated the different brain regions. Scale bar, 1 mm. **B,** Separated channels of GFP and GFAP (red), GFP was highly expressed within the olfactory bulb, cortex, hippocampus and hindbrain. Scale bar, 1 mm. **C-L,** Enlarged confocal images of different brain areas that were represented by the white dashed box in panel **A**. GFP-expressing cells were co-labeled with the astrocyte marker GFAP. (red). Scale bar, 20 μm. CB, cerebellum; HB, hindbrain; HIP, hippocampus; MB, midbrain; MO, motor cortex; OB, olfactory bulb; SS, somatosensory cortex; STR, striatum; TH, thalamus; VIS, visual cortex; PIR, Piriform cortex.

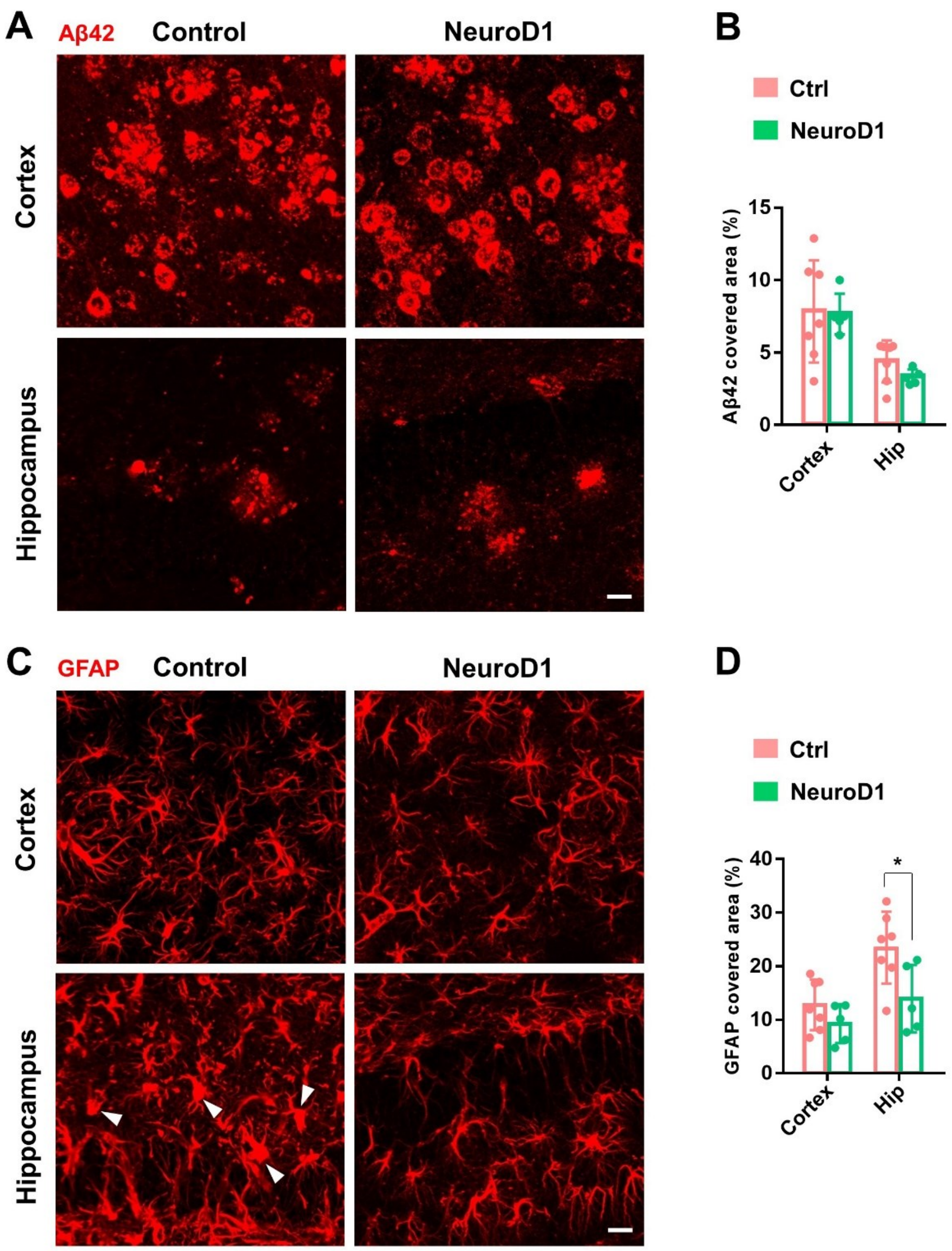

Figure S6. Aβ42 and GFAP immunostaining in 5xFAD mice. A, Aβ42 aggregates were visualized by immunostaining in cortex (top) and hippocampus (bottom). Scale bar, 20 μm. **B,** Bar graph showing there is no significant difference in the fraction of Aβ42 covered area between control and NeuroD1 groups. C, GFAP immunostaining for detecting astrocytes in 5xFAD mouse brains. Notably, some hypertrophic astrocytes were observed in 5xFAD mouse hippocampus of control group. Arrowheads indicate hypertrophic astrocytes. Scale bar, 20 μm. D, Quantified data showing GFAP covered

area were significantly reduced in 5xFAD mouse hippocampus that treated with NeuroD1. *$p < 0.05$, two-tailed Student's t-test. Data as shown mean ± SD.

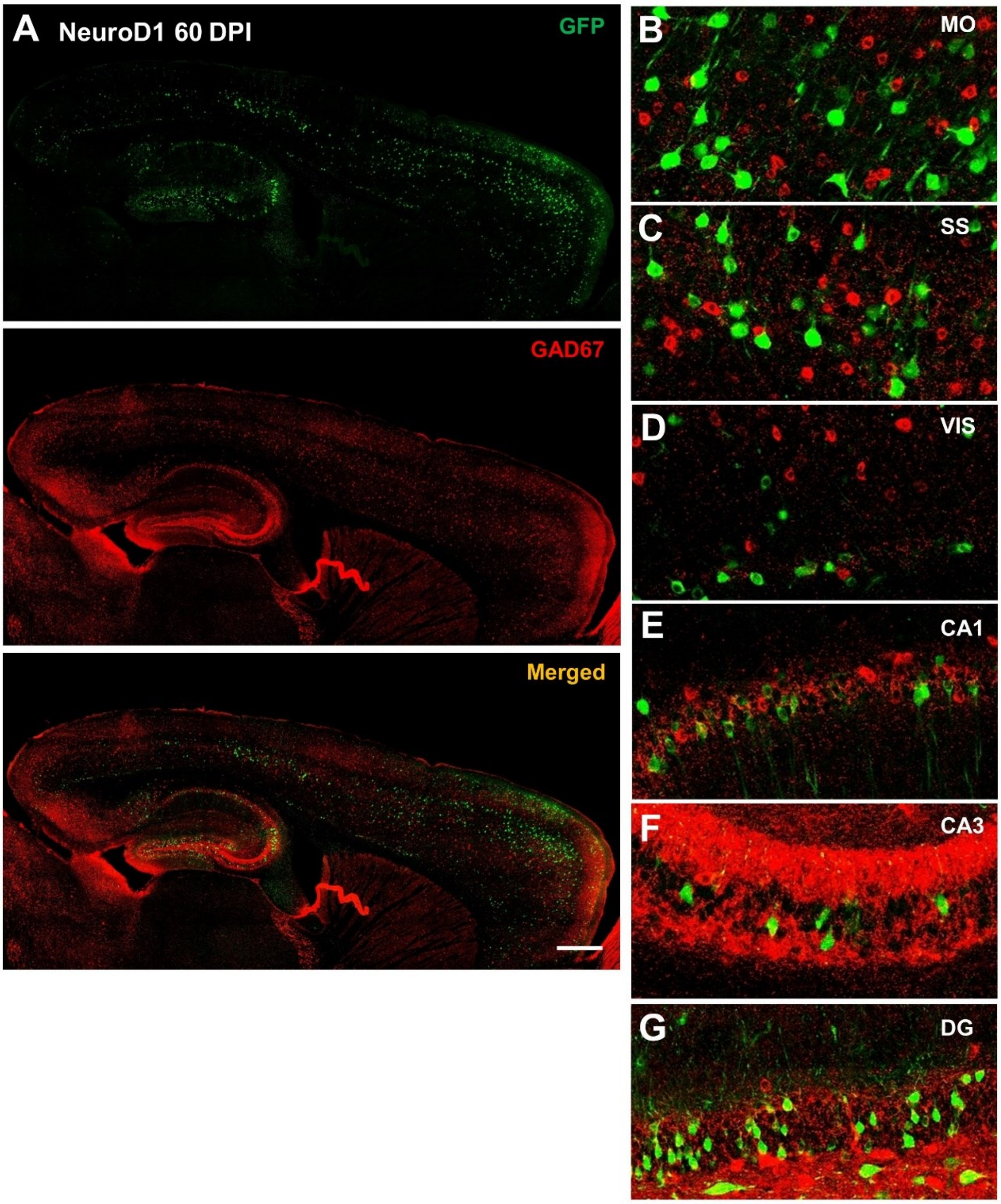

Figure S7. Converted neurons were not GABAergic. A, Low- magnification confocal images of GFP, GAD67 (red) and merged. Scale bar, 1 mm. **B-G,** High-magnification confocal images showed that GFP^+ cells in different brain regions were not co-labeled with GAD67 (red). Scale bar, 20 μm. HIP, hippocampus; MO, motor

cortex; SS, somatosensory cortex; VIS, visual cortex; CA, cornu ammonia; DG, dentate gyrus.

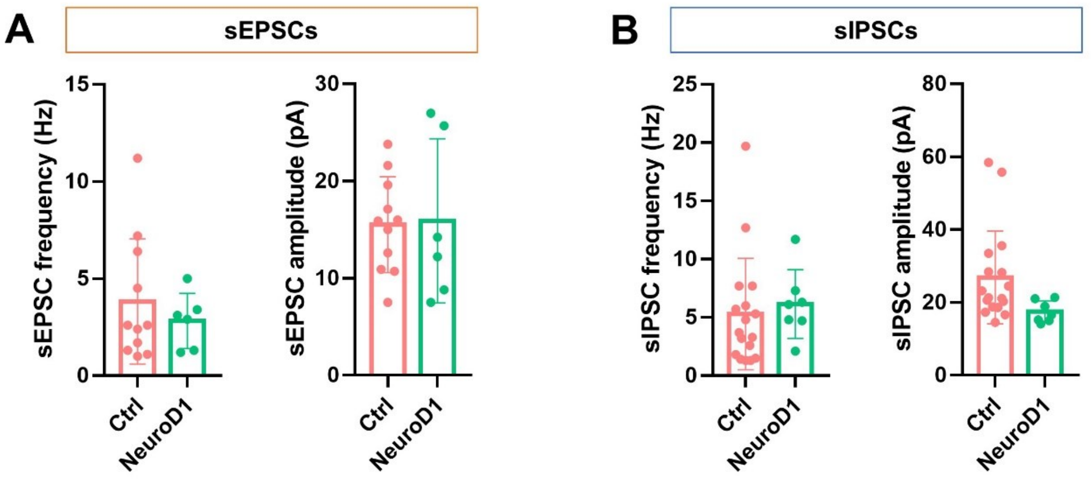

Figure S8. Spontaneous postsynaptic currents analysis of endogenous neurons in dentate gyrus. A, Quantified data of sEPSC frequency and amplitude. n = 10 for control and n = 6 for NeuroD1 group. **B,** Quantified data of sIPSC frequency and amplitude. n = 17 for control and n = 7 for NeuroD1 group. Data as shown mean ± SD.

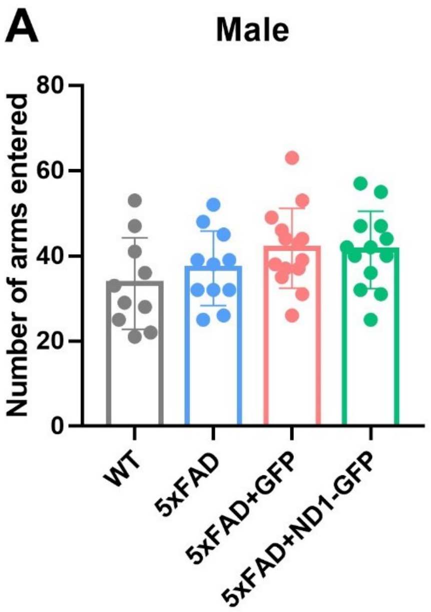

Figure S9. The total number of arms entered by mice in the Y-maze behavioral test. A, B There was no significant difference in the total number of arm entries between WT, 5xFAD, 5xFAD + GFP, and 5xFAD + NeuroD1 groups, including male (**A**) and female (**B**) mice. Data as shown mean ± SD.
